# California annual grass phenology and allometry influence ecosystem dynamics and fire regime in a vegetation demography model

**DOI:** 10.1111/nph.20421

**Published:** 2025-01-30

**Authors:** Xiulin Gao, Charles D. Koven, Marcos Longo, Zachary Robbins, Polly Thornton, Alex Hall, Samuel Levis, Stefan Rahimi, Chonggang Xu, Lara M. Kueppers

**Affiliations:** ^1^ Climate & Ecosystem Sciences Division Lawrence Berkeley National Laboratory Berkeley CA 94720 USA; ^2^ Energy & Resources Group University of California Berkeley CA 94720 USA; ^3^ Earth & Environmental Sciences Division Los Alamos National Laboratory Los Alamos NM 87545 USA; ^4^ Department of Atmospheric and Oceanic Sciences University of California Los Angeles CA 90095 USA; ^5^ National Center for Atmospheric Research Boulder CO 80301 USA; ^6^ Department of Atmospheric Science University of Wyoming Laramie WY 82071 USA

**Keywords:** annual grass phenology, California annual grassland, community structure, dynamic vegetation demography model, fire regime, grass allometry, matter and energy exchange

## Abstract

- Grass‐dominated ecosystems cover wide areas of the land surface yet have received far less attention from the Earth System Model (ESM) community. This limits model projections of ecosystem dynamics in response to global change and coupled vegetation–climate dynamics.
- We used the Functionally Assembled Terrestrial Ecosystem Simulator (FATES), a dynamic vegetation demography model, to determine ecosystem sensitivity to alternate, observed grass allometries and biophysical traits, and evaluated model performance in capturing California C_3_ annual grasslands structure and fire regimes.
- Grass allometry, leaf physiology, plant phenology, and plant mortality all drove the seasonal variation in matter and energy exchange and fire dynamics in California annual grasslands. Allometry influenced grassland structure and function mainly through canopy architecture‐mediated space and light competition instead of through carbon partitioning strategy. Regional variation in grassland annual burned area was driven by variation in ecosystem productivity.
- Our study advances the modeling of grassy ecosystems in ESMs by establishing the importance of grass allometry and plant phenology and mortality in driving C_3_ annual grassland seasonal dynamics and fire regime. The calibrated annual grass allometry and biophysical traits presented can be applied in future studies to project climate–vegetation–fire feedbacks in annual grass‐dominant ecosystems under global change.

## Introduction

Grasslands cover > 30% of the Earth surface; therefore, accurately representing grassland ecosystems in Earth System Models (ESMs) is important for understanding vegetation–climate–fire feedbacks (Blair *et al*., 2014). Grasslands also store about one‐third of global terrestrial carbon stocks, mostly in the form of soil organic matter, which may be more stable under changing climate and shifting disturbance regimes than living biomass (Bai & Cotrufo, 2022; Wilcox *et al*., 2023). Grasslands are one of the predominant vegetation types in arid and semiarid regions where tree cover is limited by climate and recurrent disturbances (Anderson, 2006). Persistence of grasses in ecosystems such as grasslands and savannas depends on just‐enough precipitation and periodic disturbances to prevent woody plant encroachment and maintain a dynamic equilibrium (Scholes & Archer, 1997; Marañón *et al*., 2009). However, anticipated changes in the frequency and intensity of precipitation extremes and fire disturbances will likely alter species composition and thus ecosystem structure and carbon dynamics in grasslands (Staver *et al*., 2011; Yu *et al*., 2017; D'Onofrio *et al*., 2019). Yet, representing change in these grassy ecosystems in ESMs remains a modeling challenge due to the complexity introduced by climate–vegetation–fire feedbacks and limited investment in simulating herbaceous communities (Beckage *et al*., 2009, Dantas *et al*., 2016, Holdo & Nippert, 2023).

In the last decade, dynamic vegetation demography models (VDMs) that capture size‐dependent growth, mortality, and competition for water, nutrients and light have been a focus of development by the ESM community to better predict the role of vegetation dynamics on global carbon cycles (Fisher *et al*., 2018). They are also useful tools for understanding the local and regional drivers of community structure and ecosystem function. However, most vegetation demographic models (e.g. LPJ‐GUESS, ED2, and FATES but see aDGVM) were originally developed for closed‐canopy forests with most model applications hitherto focused on tree‐dominated systems, resulting in less developed model processes and poorly calibrated model parameters for grass plant functional types (PFTs) and open ecosystems (Sitch *et al*., 2003; Medvigy *et al*., 2009; Moncrieff *et al*., 2014; Koven *et al*., 2020). One of the fundamental differences between trees and grasses is the size‐dependent carbon allocation to different plant structures (Niklas, 2004), which is important for understanding plant–environment interactions and species competition (Shipley & Meziane, 2002; Metcalf *et al*., 2006; McCarthy & Enquist, 2007). For instance, greater allocation to stem biomass enables greater access to light, while greater allocation to deep root biomass enables access to groundwater to help plants avoid drought and effectively compete for water compared to shallowly rooted plants (Holmes & Rice, 1996). Due to the lack of empirical data on individual grass biomass allocation and plant architecture, parameterization of grass allometry (hereafter referring to the size dependence of biomass, allocation, and canopy architecture) in these VDMs is less constrained or does not distinguish between different grass functional types (e.g. C_3_ vs C_4_ and annual vs perennial grass) despite known species differences in growth and development (Sitch *et al*., 2003; Medvigy *et al*., 2009; Nafus *et al*., 2009).

However, differences in biomass allocation can potentially influence ecosystem structure, function, and fire regime through partitioning of net primary productivity to aboveground biomass, and thus fuel load, or between photosynthetic and supporting structures to influence carbon assimilation and transport (Li *et al*., 2018). Variations in plant canopy architecture can also influence community structure: A larger crown area may result in reduced stem density per unit ground area due to stronger competition for space and light (Pretzsch *et al*., 2012). Interactions between biomass partitioning and canopy architecture and the resulting impacts on community structure and ecosystem functioning are not clear. In addition, some grasses are annual plants that differ from perennials in terms of leaf phenology and plant life span, which both influence the seasonal variation in matter and energy exchange and fire regime (Davies & Nafus, 2013). Yet, most VDMs assume grass PFTs to be perennials (Bart *et al*., 2017). Misrepresenting grass life history, allometry and phenology in vegetation demographic models can generate unrealistic biomass patterns in grasslands, which in turn may affect how the model simulates community structure, fire behavior, and vegetation–fire feedbacks (Wilcox *et al*., 2023).

Grasses in Mediterranean regions are mainly annual species that are adapted to seasonal droughts by completing reproduction before the onset of the dry season and persisting as dormant seeds until the first rainfall in early winter (Fernández Ales *et al*., 1993; Volis *et al*., 2002; Sherrard & Maherali, 2006). Seasonal dynamics in matter and energy exchange, which are driven by the phenology and life history of the dominant species, thus coincide with the seasonal variation in soil water content in these annual grasslands (Xu & Baldocchi, 2004; Liu *et al*., 2011). Grasses are also the main components of surface fuels in open ecosystems, influencing the regional fire regime (Vilà *et al*., 2001; Rahlao *et al*., 2009; Balch *et al*., 2013). Change in grass phenological phase therefore directly influences wildfire dynamics: As the curing level of grass fuels increases, fire risk increases due to decreased fuel moisture (Wittich, 2011; Cruz *et al*., 2015). Capturing annual grass phenology, life span and fuel conditions, and their responses to climate variability is important for reconstructing the historical fire regime and projecting community dynamics into the future for these fire‐prone ecosystems.

Using modeling experiments with generalized and species‐specific grass allometries and varying plant traits, we addressed the following research questions: (1) how does variation in biomass partitioning between leaf and stem, and in canopy architecture, influence simulated ecosystem structure and function in California annual grasslands? (2) What plant traits and ecological processes are important in controlling the mean state in ecosystem properties and fire behavior, and how does trait importance to ecosystem properties change seasonally? We also use site‐optimized parameters to run the model across California annual grasslands, validating model performance and determining the main drivers for grassland annual burned area. We hypothesize that: (1) a higher carbon partitioning to leaf than to stem will result in a more productive ecosystem despite the variations in canopy architecture; (2) traits controlling leaf physiology, plant phenology, and sensitivity to soil moisture are important in controlling ecosystem energy and matter exchange and their seasonal variations; (3) fire behavior is determined by both fuel traits and key ecological processes such as mortality and litter decomposition; and (4) variation in ecosystem productivity drives annual burned area at the regional scale.

## Materials and Methods

### Vegetation model

**Fig. 1:**
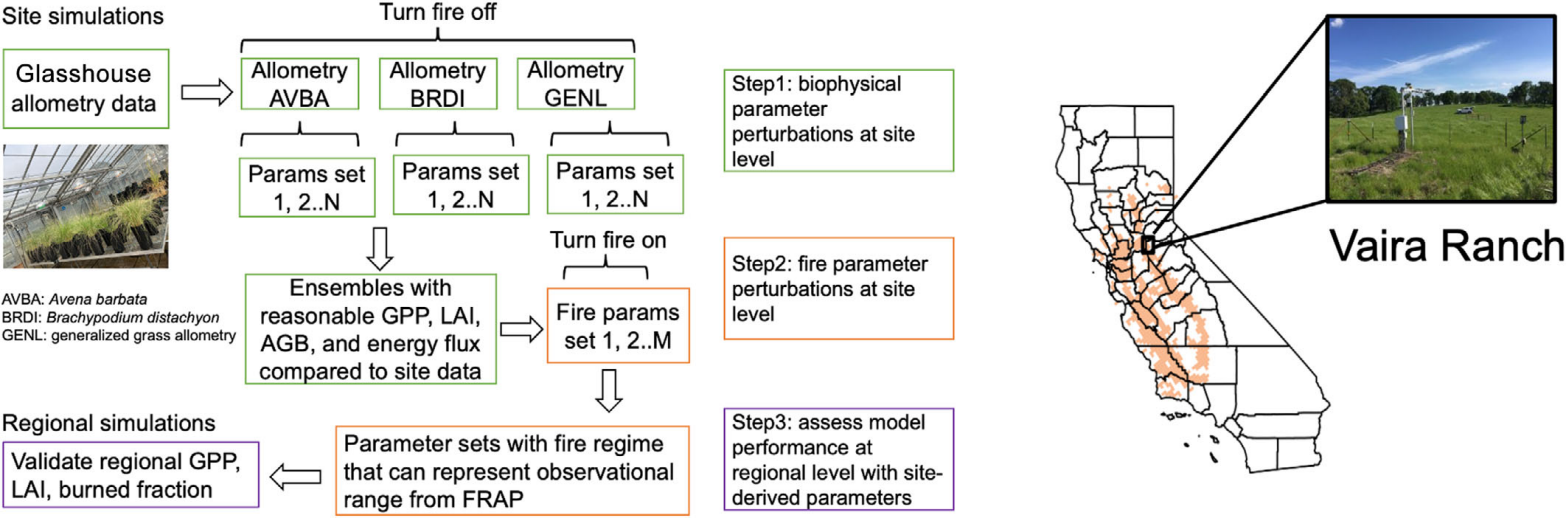
Model experimental design, study site and region. Site‐level parameter perturbation experiments were conducted at Vaira Ranch, California, USA. Evaluation of model performance was conducted across the California annual grassland region, indicated by the orange grid cells in the map.

To address our questions, we used a VDM: the Functionally Assembled Terrestrial Ecosystem Simulator (FATES) coupled to the Community Land Model (CLM), running simulations at a single site and across the entire California annual grassland region (Fig. 1). FATES is a cohort‐based, vegetation demography model that incorporates plant competition for resources, multiple disturbance types, and simulates the climate–vegetation–fire feedback when coupled to land and atmospheric models (Koven *et al*., 2020). FATES tracks ecosystem dynamics by resolving PFT competition for light, space, and water (Fisher *et al*., 2015, 2018). Plant architecture and carbon allocation to different plant organs follow PFT‐specific allometric relationships defined by a set of model parameters. Plant mortality is driven by disturbances such as drought and fire and self‐thinning processes. Spatial heterogeneity in community structure is represented by cohorts varying in diameter and height, which occur on patches varying in time‐since‐last‐disturbance. FATES represents plant phenology in three ways: evergreen, drought‐, or cold‐deciduous leaf habit. Environmental factors such as daily mean temperature and soil water content aggregated across the root profile are used, along with user‐defined plant response thresholds, to determine the timing of leaf‐on and leaf‐off. We used the drought‐deciduous phenology to simulate Mediterranean annual grasses that respond to seasonal change in soil water content, and relied on drought‐driven mortality to regulate the population size, and therefore leaf area and live biomass of grasses at different times of the year.

FATES uses an approach based on the SPITFIRE model (Thonicke *et al*., 2010) to track fuel dynamics, fire risk, fire behavior, and the resulting fire damage and plant mortality (Shuman *et al*., 2024). Fuel load and fuel moisture content are the two key fuel traits influencing fire behavior and fire effects. Fuel is calculated as the sum of live grass biomass, leaf litter, and aboveground coarse woody debris excluding 1000‐h fuels (dead woody fuels with diameter > 7.6 cm). Fuel moisture content is either a function of fire weather and drying rate for dead fuels; or is determined by soil water content of the top 30 cm of soil for live grass fuels. Patch‐level fuel moisture content is then calculated as the fuel‐load‐weighted average fuel moisture content of dead and live grass fuels. Fuel characteristics such as surface area to volume ratio and fuel bulk density play important roles in determining rate of spread, which in turn influences fire intensity and burned area. As most perennial grasses resprout after fire (Simpson *et al*., 2022), currently in FATES, there is no fire‐induced mortality for grass PFTs. However, burning of aboveground biomass by fire can deplete the carbon storage pool required to regrow the burnt tissues, and thus, fire impacts can be indirectly reflected as part of carbon starvation mortality.

FATES needs to be coupled to a host land model to simulate the soil hydrology and canopy energy balance. Currently, FATES is readily coupled to the CLM5 (Lawrence *et al*., 2019; Buotte *et al*., 2021) and the E3SM land model (Golaz *et al*., 2019; Ma *et al*., 2021). In this study, we used CLM5 as the host land model, and we refer to the model hereafter as CLM–FATES.

### Study region and available observations

We conducted site‐level simulations at Vaira Ranch (38.4133° N, 120.9508° W), a C_3_ annual grassland with < 10% tree cover located in the lower foothills of the Sierra Nevada Mountains, California, USA. Mean annual precipitation at the site is 560 mm, mostly occurring between October and May (Baldocchi *et al*., 2004). The plant community is dominated by C_3_ annual grasses including *Brachypodium distachyon* L., *Bromus madritensis* L., and *Avena* spp. (Xu & Baldocchi, 2004). The soil is classified as a rocky silt loam with 13% clay and 30% sand (Baldocchi *et al*., 2004). To drive and benchmark site‐level simulations, we used the flux tower measurements from 2000 through 2014 (Ma *et al*., 2022). Ecosystem measurements are available for evaluating simulated gross primary productivity (GPP), leaf area index (LAI), live aboveground biomass (AGB), and energy fluxes (Ma *et al*., 2022).

We ran regional simulations across grid cells with noncrop herbaceous cover ≥ 80% as determined by the National Land Cover Database (Homer *et al*., 2004). To assess the CLM–FATES representation of vegetation structure and carbon cycle, we compared model output with remote sensing estimates of GPP (Xiao *et al*., 2014) and LAI (Lin *et al*., 2023). Both GPP and LAI data were resampled to the model domain and spatial resolution, which is 9 km. Burned area data during the 2000–2020 period collected by The California Department of Forestry and Fire Protection's Fire and Resource Assessment Program (FRAP) were used to assess simulated burned fraction (Fire and Resource Assessment Program. Fire Perimeters 2020). We first calculated the fraction of each model grid intersected by the burned area polygon each year during 2000–2020. We then masked the regional burned fraction using the derived grassland mask to retain burned fraction each year only for grassland regions.

### Parameter perturbation experiments and model validation

We carried out two parameter perturbation experiments at the site level for leaf traits and phenology, drought tolerance, drought‐induced mortality, and fuel characteristics (Table 1), considering three alternate allometry assumptions.

**Table 1:** Model parameters examined in this study and the minimum and maximum values used for parameter perturbation experiments.

Category	Parameter short	Parameter description	Min	Max	Source
Physiology	Leaf_Dia_	Leaf diameter (m)	0.01	0.04	Prior simulations
	Leaf_Turnovr_	Leaf turnover (yr^−1^)	0.02	0.32	TRY
	SLA_top_	Specific leaf area (m^2^ g^−1^ C)	0.015	0.072	TRY
	$V_{c_{\max}}$	Maximum carboxylation rate (μmol CO_2_ m^−2^ s^−1^)	35.6	91.6	Maire *et al*. (2012); Griffith *et al*. (2020)
	BB_incpt_	Stomatal intercept (μmol H_2_O m^−2^ s^−1^)	10 000	2030 000	Miner *et al*. (2017)
	BB_slope_	Stomatal slope (unitless)	5.25	17	Miner *et al*. (2017)
	LNC	Leaf N : C ratio (leaf g N leaf g^−1^ C)	0.01	0.06	TRY
Reproduction	Recruit_DBH_	Reproduction basal diameter threshold (cm)	1.5	4	Gao *et al*. (2024)
	Recruit_Alloc_	Seed allocation (mature, fraction)	0.1	1	Prior simulations
	Recruit_hgt_	Recruitment min. height (m)	0.1	0.5	Prior simulations
	C_Storage_	Storage allocation (fraction)	1	1.5	Prior simulations
Phenology	SWC_Phen_	Soil water content drought threshold deciduous phenology (m^3^ m^−3^)	0.1	0.23	Baldocchi *et al*. (2004)
	smpsc	Soil matric potential at full stomatal closure (MPa)	−2.0	−0.6	Prior simulations
	smpso	Soil matric potential at full stomatal opening (MPa)	−0.6	−0.33	Prior simulations
	Root_a_	Rooting depth parameter a (unitless)	5	13	Schenk and Jackson (2002)
	Root_b_	Rooting depth parameter b (unitless)	3	10	Schenk and Jackson (2002)
Mortality	SM_hydro_	Soil moisture (drought mortality begin, unitless)	0.25	0.9	Prior simulations
	Mort_hyd‐scala_	Hydraulic mortality scalar (yr^−1^)	3	20	Prior simulations
	Mort_cst‐scala_	Carbon starvation mortality scalar (yr^−1^)	1	6	Prior simulations
	Respir_grow_	Growth respiration (unitless)	0.1	0.5	Prior simulations
Fire	FBD_dead_	Fuel bulk density (dead, kg m^−3^)	4	22	Prior *et al*. (2017); Snell (1979)
	FBD_live_	Fuel bulk density (live, kg m^−3^)	1	4	Snell (1979)
	Ignition	Ignition density (strikes km^2^ yr^−1^)	0.01	1	Keeley & Syphard (2019)
	DryingRatio	Fuel drying ratio (unitless)	66	66 000	Prior simulations
	FuelEnergy	Fuel energy (kJ kg^−1^)	6450	14 300	Simpson *et al*. (2022)
	MaxDecomp	Maximum litter fragmentation rate (g g^−1^ yr^−1^)	0.8	1.6	Zhang *et al*. (2008)

To parameterize allometry in FATES, which is defined by 22 interrelated model parameters that govern the relationship between plant diameter and height, crown area, and tissue‐specific biomass pools, we used observed species‐specific allometric relationships for two C_3_ annual grasses that are dominant species at the site, *Brachypodium distachyon* (BRDI) and *Avena barbata* Pott ex Link (AVBA), and a general C_3_ annual grass (GENL) allometry using data pooled from four common California C_3_ annual grasses (Gao *et al*., 2024). We chose the two species‐specific allometries to represent strong differences in canopy architectures (e.g. AVBA represents a tall grass with a wide crown area, while BRDI represents a short grass with a narrow crown area) and carbon partitioning between leaf and stem for the dominant species at the study site (Supporting Information Fig. S1). In FATES, for all PFTs, plant size is tracked using a diameter variable, which serves as the independent variable for all allometric relationships. For trees, this diameter corresponds to the stem diameter at breast height (DBH); for grasses, we use basal diameter of the grass as the index variable. Grass PFTs do not produce woody biomass or fuels; all aboveground grass biomass eventually goes into a grass litter pool after mortality. The details of how we defined each allometric relationship are provided in Notes S1; Table 2.

**Table 2:** Model allometry parameters and the corresponding values for *Avena barbata*, *Brachypodium distachyon*, and generalized allometry used in this study, using data from Gao *et al.* (2024). The letters a, b, and c indicate corresponding model parameters for each applied allometry model.

Parameter	AVBA	BRDI	GENL	Allometric equation
AGB allometry coefficient	0.00368612	0.02353439	0.00206078	AGB = *c* × height^a^ × BD^b^
AGB allometry exponent for height	1.463894	2.20989	1.209238	
AGB allometry exponent for DBH	0.9188281	1.0510878	1.614535	
Leaf allometry coefficient	0.00041331	0.00044499	0.000434	Leaf = *a* × min (BD, BD_max.hgt_)^b^
Leaf allometry exponent for DBH	2.090633	1.597315	1.915118	
Height allometry coefficient	0.08811922	0.12812118	0.1476171	Height = *a* × min (BD, BD_max.hgt_)^b^
Height allometry exponent for DBH	1.465103	0.510092	0.6995105	
BD at maximum plant height (cm)	7	9	9	
Leaf to fine root biomass ratio	1	1	1	Froot = *a* × Leaf
Coefficient for crown area allometry	0.03220912	0.01651188	0.02913376	Crown area = *a* × BD^b^
Difference between crown area and leaf biomass allometry exponents for DBH	−0.720075	−0.182835	−0.671656	
Intercept for the leaf area – stem area relationship	1000	1000	1000	
Baseline seed reproduction allocation before reach the threshold reproductive size	0	0	0	
Initial recruitment density for bare ground start	100	100	100	

AGB, aboveground biomass; BD, basal diameter for grasses; BD_max.hgt_, basal diameter at which the plant reaches its maximum height; DBH, diameter at breast height; Froot, fine root biomass.

For all other parameters, the minimum and maximum values of each model parameter, obtained either from the literature or prior model simulations, were used to define the parameter space for Latin hypercube sampling (McKay, 1992). To account for potential parameter correlations in Latin hypercube sampling, we built a Spearman rank correlation matrix based on the available C_3_ grass trait data from the TRY database (Kattge *et al*., 2020), and only accounted for correlations that were statistically significant at the 95% confidence level (*P* < 0.05) to avoid introducing spurious correlations. We sampled values for 20 parameters related to nonallometry traits (Table 1) from uniform distributions and significant rank correlations, generating 1500 ensemble members. To determine the effects of grass allometry on ecosystem structure and function, we repeated the 1500‐member ensemble three times, once for each of three grass allometries. This experimental design resulted in three 1500‐member ensembles with allometry differing between the three ensembles to answer the first question; and nonallometry traits varying between the 1500 members within each ensemble to answer the second question. To facilitate comparison to observations, this first parameter perturbation experiment was conducted under a no‐fire scenario because there was no fire recorded at Vaira Ranch during the observation period.

Ensemble member simulations were then compared with observations and selected if simulated GPP, LAI, AGB, latent, and sensible heat flux simultaneously captured the observed seasonal variation, with model monthly means during the growing season within 15–85% quantiles of the observations in the same month. The 15–85% quantiles were chosen to avoid overfitting the model at the site level. This is necessary because when applying site‐derived parameters to regional simulations, spatial heterogeneity in climate and soil properties will likely result in a wide range of ecosystem structure and functioning, which a strictly constrained parameterization based on the mean state of one single site may fail to capture. A total of eight ensemble members, which spanned our three allometry assumptions, met our criteria and formed the basis for the second site‐scale parameter perturbation experiment.

Our goal with the second parameter perturbation experiment was to understand how fire‐relevant parameters influence simulated fire behavior, and to tune those parameters to where the simulated fire area falls within the range of observed burned area across California annual grasslands. Six fire‐relevant parameters were varied to determine simulated fire area sensitivity (Table 1). Those parameters were chosen based on previous work focusing on woody fuels (Buotte *et al*., 2021). Using each of the eight PFT parameter sets selected from the fire‐off experiment and holding parameters besides the six fire parameters constant within the ensemble, we turned on the fire dynamics and ran CLM–FATES with 500 new ensemble members. We again filtered the ensemble members, retaining those that represented ecosystem characteristics. We also calculated the 15 and 85% quantiles for annual burned fraction across all grassland grid cells in California for Years 2000–2020 and retained parameter sets for which more than half of the simulated years were within this range. The observations are positively skewed, with many years having zero burned area due to both the discrete nature of wildfires and active fire suppression. We thus used these filtering criteria to capture the central tendency and the interannual variability of the observations without requiring simulated burned area to reach zero in low fire years given that CLM–FATES treats burned area as a continuous rather than discrete phenomenon and does not yet include a fire suppression process.

All parameter perturbation simulations used 2000–2014 meteorological data from the flux tower at Vaira Ranch to drive the model for 80 model years to reach an equilibrium biomass state by recycling the forcing data, with CO_2_ concentration fixed at 400 ppm, starting from a bare ground state. Current default trait values for grass PFTs in FATES are either inherited from other vegetation demography models (e.g. ED2) or based on observations from woody plants, and have not yet been thoroughly validated for Mediterranean ecosystems. Therefore, we also used the same meteorological drivers, but default C_3_ grass parameters, to demonstrate the default California grassland prediction.

To understand the sensitivity of ecosystem structure and function to allometry and nonallometry traits, we fit linear regression models for the time‐means of simulated GPP, LAI, AGB, total latent heat flux, and burned area as functions of the allometry group that defines each ensemble (as a discrete variable) or one of the continuously varying traits that defines each member within an ensemble, and calculated the percentage of variation that was explained by allometry group or each trait for each model variable. The importance of plant traits was then ranked by the percent variance explained for each simulated variable. To determine how plant trait effects change seasonally, we grouped model results into growing (January to April), senescence (May to August), and emergence (September to December) seasons, calculated model means for each season and conducted sensitivity analyses for each following the same procedure as discussed earlier.

### Regional simulation and model evaluation

Finally, we ran CLM–FATES with three final selected parameter sets across California annual grasslands and compared model simulations of regional GPP, LAI, and burned fraction to observations. To drive the CLM–FATES simulations, we used 1981–2020 ERA5 reanalysis data, dynamically downscaled to 9 km for California (Rahimi *et al*., 2022). We followed the same protocol to run the regional simulations as for running the site simulations. Model output for the last 20 yr of the simulations (2000–2020) were used for model performance evaluation by comparing simulated annual mean to the corresponding annual mean of each observed variable and calculating the root‐mean‐squared error (RMSE). We then determined the major drivers of annual burned area by doing sensitivity analysis as described above.

## Results

### Grass allometry influences ecosystem structure and functioning

**Fig. 2:**
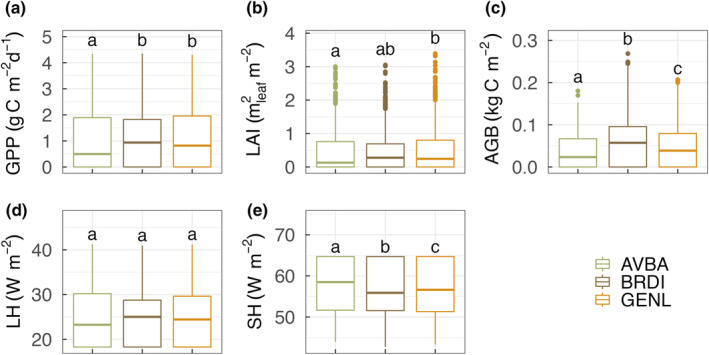
Allometry effects on model simulated carbon, water, and energy flux at the site. Model simulated (a) gross primary productivity, (b) leaf area index, (c) aboveground biomass, (d) latent heat flux, and (e) sensible heat flux varied between the three allometry groups (significant differences at *P* < 0.05 between groups using ANOVA test are indicated by different letters). Allometry groups are color‐coded: AVBA refers to the *Avena barbata* allometry, BRDI refers to the *Brachypodium distachyon* allometry, and GENL refers to the generalized C_3_ annual grass allometry. Variation within each allometry group is due to the difference in 20 additional nonallometry biophysical traits that were varied among ensemble members. From the bottom to the top of each box, the horizontal line refers to the first quantile, median value, and the third quantile of model simulated mean; lower whiskers refer to the minimum value and upper whiskers refer to the maximum value of model simulated mean respectively; points are outliers.

Our results showed that simulated carbon, water, and energy fluxes were strongly affected by the choice of allometry in the model. The BRDI ensemble had the highest model mean GPP (1.13 ± 1.07 g C m^−2^ d^−1^), AGB (0.06 ± 0.06 kg C m^−2^), and latent heat flux (25.06 ± 5.76 W m^−2^), but the lowest average sensible heat flux (56.67 ± 6.25 W m^−2^), which is in contrast to the AVBA ensemble (GPP: 0.98 ± 1.14 g C m^−2^ d^−1^, AGB: 0.04 ± 0.04 06 kg C m^−2^, latent heat flux: 24.7 ± 6.9 W m^−2^, Fig. 2). The GENL ensemble had results that were intermediate between the BRDI and AVBA ensembles except that the GENL ensemble had the highest mean LAI (0.48 ± 0.6 m^2^ m^−2^) while the AVBA ensemble had the lowest mean LAI (0.42 ± 0.56 m^2^ m^−2^). The effects of grass allometry on model predictions were strongest for AGB and sensible heat flux, where we found statistically significant differences across the three ensembles (*P* < 0.001).

### Effects of biophysical traits on matter and energy exchange and their seasonal dynamics

**Fig. 3:**
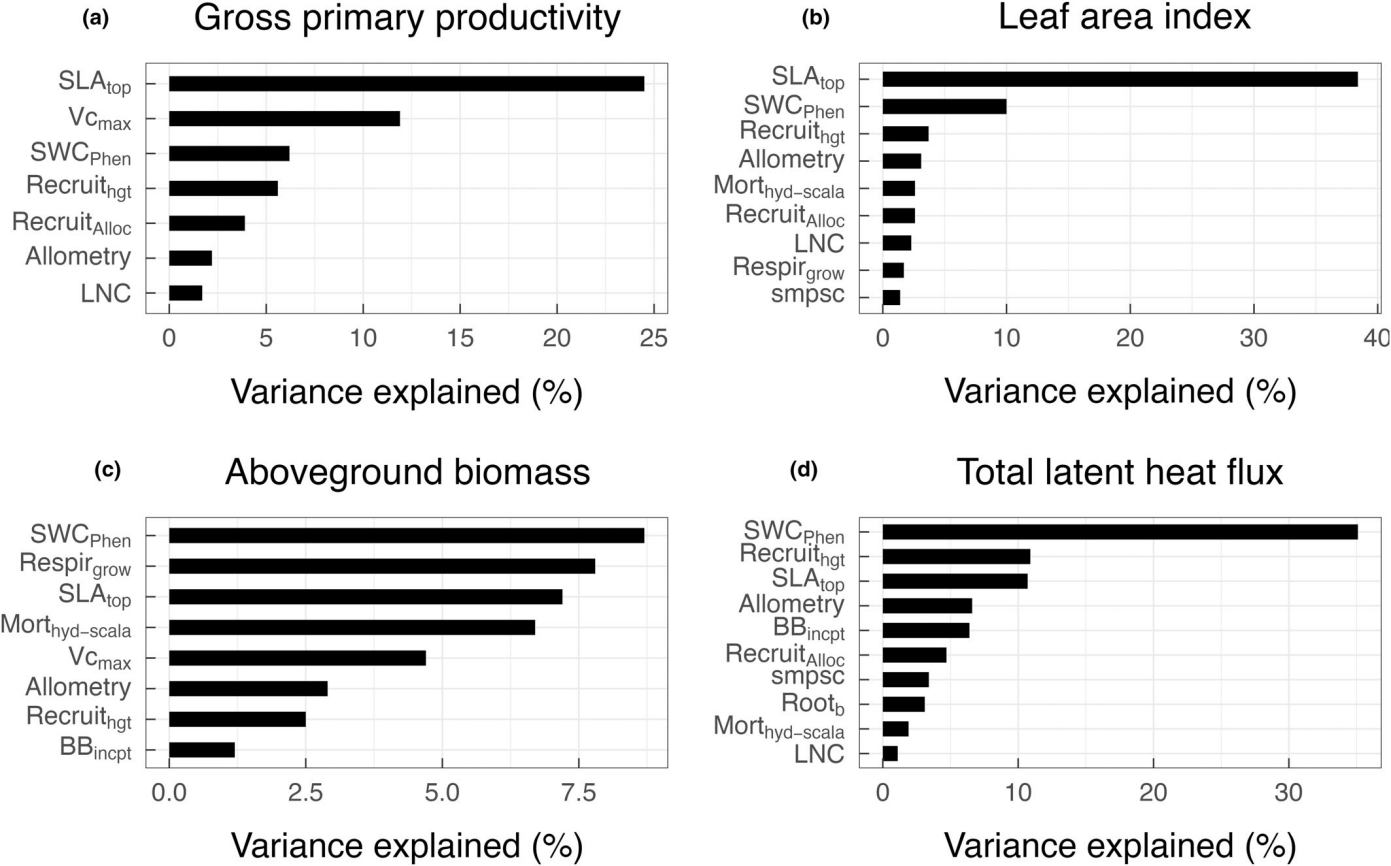
Model sensitivity to examined parameters. Important model parameters for simulated gross primary productivity (a), leaf area index (b), aboveground biomass (c), and total latent heat flux (d). Parameters are ranked in descending order by percent variance explained by each parameter for the corresponding model variable. BB_incpt_, Ball–Berry stomatal conductance intercept; LNC, leaf N : C ratio; Mort_hyd‐scala_, drought‐induced mortality rate; Recruit_alloc_, allocation to reproduction once plants reached the threshold reproductive size; Recruit_hgt_, height of new recruitment; Respir_grow_, growth respiration rate; Root_b_, parameter b for rooting depth profile; SLA_top_, specific leaf area at top of the canopy; smpsc, threshold soil matric potential for stomatal closure; SWC_Phen_, threshold soil water content triggering drought‐deciduous phenology; $V_{c_{\max}}$, maximum carboxylation rate at 25°C.

Carbon, water, and energy fluxes and their seasonal dynamics were mainly influenced by plant leaf traits and phenology in CLM–FATES. Across all simulations, specific leaf area (SLA) alone explained 24.5% and 38.4% of the total variation in GPP (1.06 ± 2.26 g C m^−2^ d^−1^) and LAI (0.45 ± 0.85 m^2^ m^−2^), respectively (Fig. 3). For AGB (0.047 ± 0.07 kg C m^−2^) and total latent heat flux (24.86 ± 22.39 W m^−2^), the soil water content threshold triggering leaf‐on and leaf‐off (SWC_Phen_) was the most important trait, explaining 8.7% and 35.1% of total variation in the two model outputs, respectively. Among these four model variables, AGB was the least sensitive to trait perturbations. In addition to SLA and SWC_Phen_, the maximum leaf carboxylation rate ($V_{c_{\max}}$), plant height of new recruits, and growth respiration cost were among the top three important traits influencing simulated matter and energy exchange at the site (Fig. 3). In general, SLA was positively correlated with the four model variables, while SWC_Phen_ was negatively correlated (Fig. S2).

**Fig. 4:**
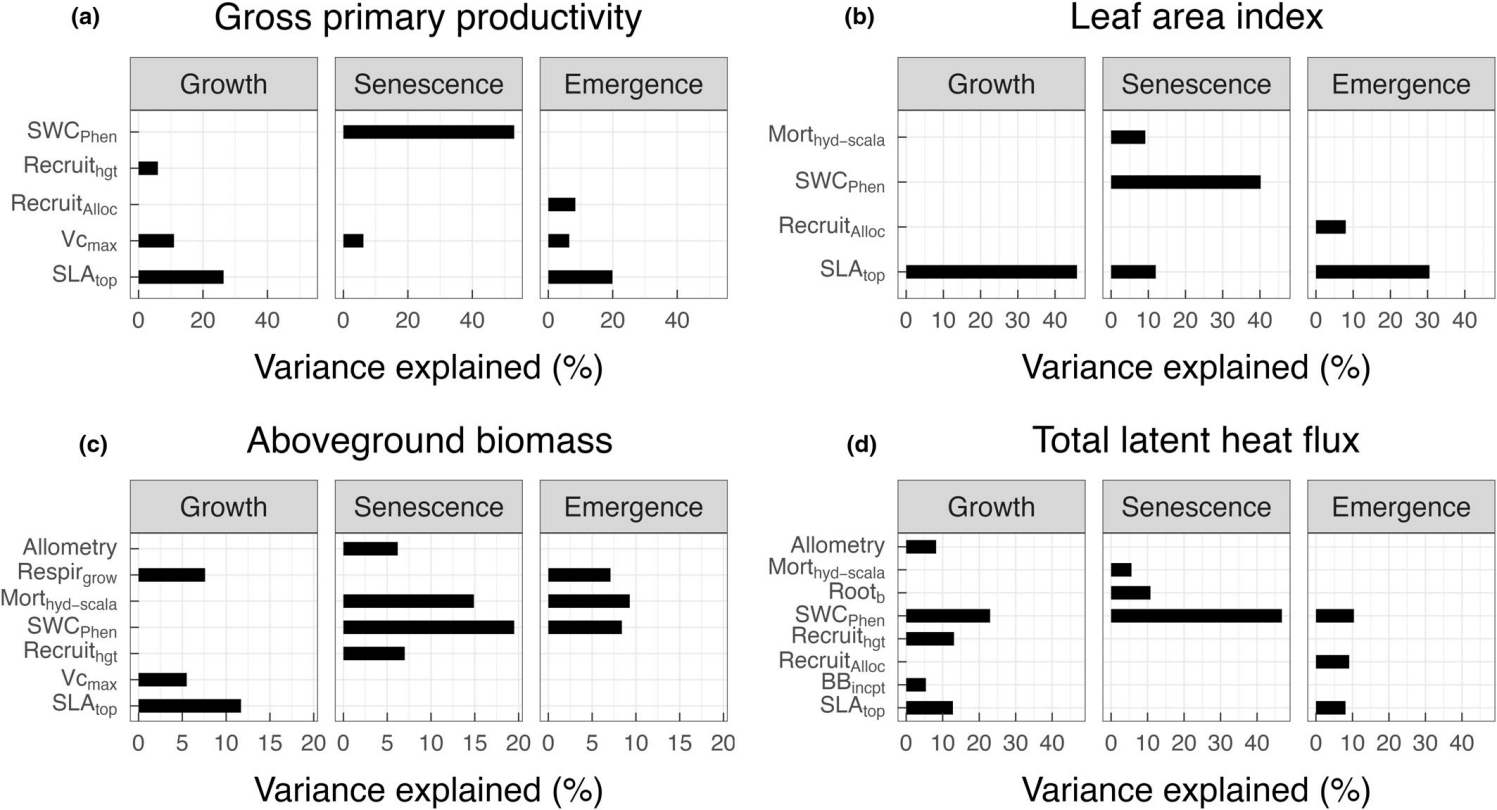
Difference in parameter importance as grass phenological phase changes. The top Community Land Model–Functionally Assembled Terrestrial Ecosystem Simulator model parameters determining at least 5% of the variance in simulated gross primary productivity (a), leaf area index (b), aboveground biomass (c), and latent heat flux (d) varies as the phenological phase of the grass plant functional type changes from growth to senescence to dormancy. BB_incpt_, Ball–Berry stomatal conductance intercept; LNC, leaf N : C ratio; Mort_hyd‐scala_, drought‐induced mortality rate; Recruit_alloc_, allocation to reproduction once plants reached the threshold reproductive size; Recruit_hgt_, height of new recruitment; Respir_grow_, growth respiration rate; Root_b_, parameter b for rooting depth profile; SLA_top_, specific leaf area at top of the canopy; smpsc, threshold soil matric potential for stomatal closure; SWC_Phen_, threshold soil water content triggering drought‐deciduous phenology; $V_{c_{\max}}$, maximum carboxylation rate at 25°C.

The relative importance of traits determining simulated GPP, LAI, and AGB differed according to the phenological phase of the grass PFT. SLA was the key plant trait influencing GPP and LAI throughout the growth stage (January to April) and later during the emergence stage (September to December), when plants were actively growing or slowly greening up (Fig. 4). By contrast, SWC_Phen_ was the top trait during the senescence stage (May to August) when plants were browning‐down during the dry season (Fig. 4). A similar pattern was observed for simulated AGB, except that mortality rate due to hydraulic failure was the dominant trait affecting AGB during the emergence stage, suggesting a lagged response of AGB to drought‐induced mortality in established plants. SWC_Phen_ remained as the most important trait influencing total latent heat flux throughout the three phenological phases.

**Fig. 5:**
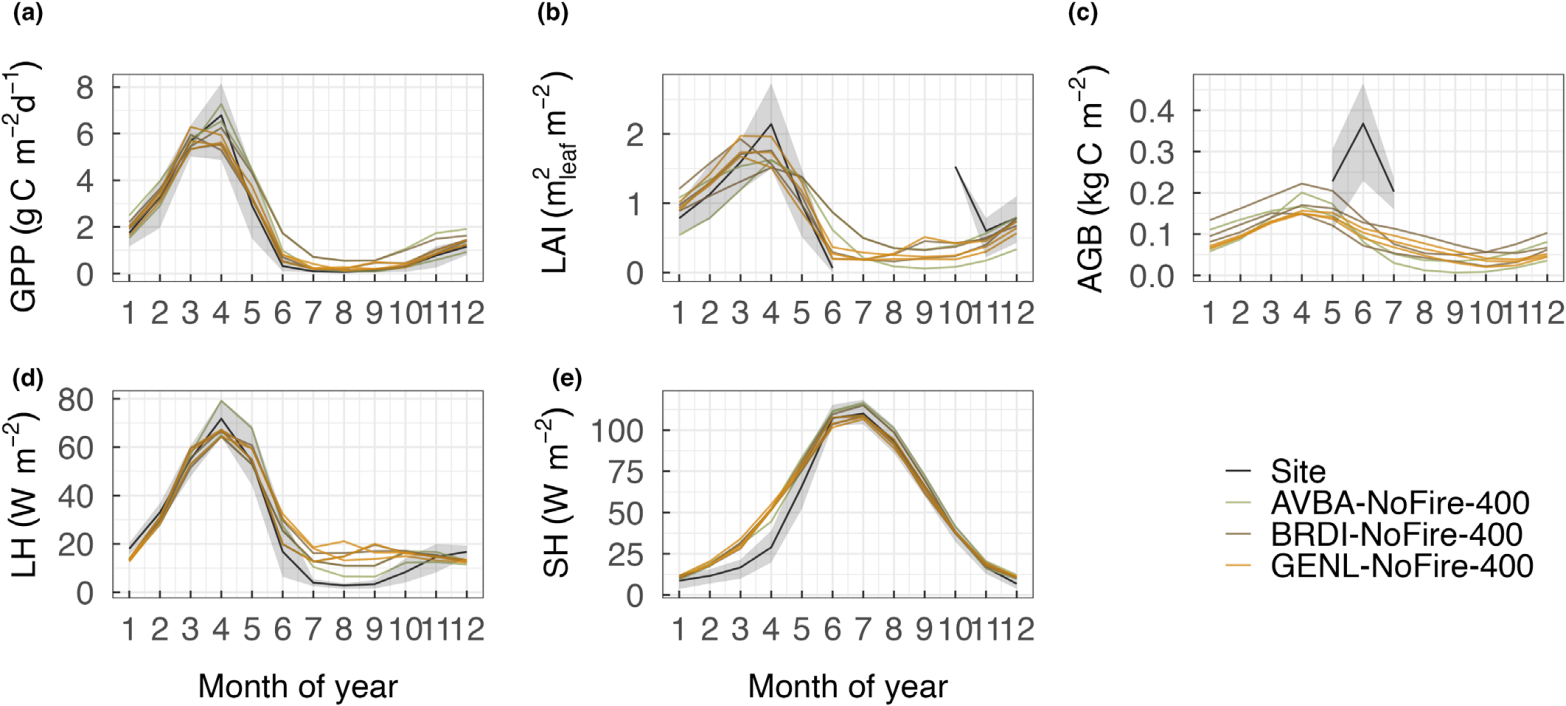
Community Land Model–Functionally Assembled Terrestrial Ecosystem Simulator simulated carbon, water, and energy fluxes and their seasonal dynamics compared to observations at Vaira Ranch. Model monthly means of gross primary productivity (a), leaf area index (b), aboveground biomass (c), latent heat flux (d), and sensible heat flux (e) are only shown for the eight top‐performing ensemble members from the three allometry groups. Shaded areas are 15–85% quantiles of the mean for site observations. Ensemble members are color‐coded by allometry groups: AVBA‐NoFire‐400 refers to ensembles from the AVBA allometry group with SPITFIRE off and fixed at 400 ppm CO_2_; BRDI‐NoFire‐400 and GENL‐NoFire‐400 refer to the BRDI allometry and the generalized C_3_, annual grass allometry.

We compared model simulations with site‐level observed GPP, LAI, AGB, and energy fluxes, and retained eight parameter sets that enabled CLM–FATES to capture seasonal variation in matter and energy exchanges at the site (Fig. 5). CLM–FATES using the default, perennial C_3_ grass parameterization resulted in large model‐observation discrepancy in monthly mean GPP (Fig. S3). Selected parameter sets were from all three allometry groups with two AVBA, three BRDI, and three GENL ensemble members, respectively. Simulated mean GPP and sensible heat flux varied less across the eight ensemble members than did LAI, AGB, and latent heat flux in the dry season (Fig. 5). In addition, divergence between model simulations and observations was largest for LAI and AGB; selected ensemble members tended to overestimate the former and underestimated the latter.

### Fuel traits and ignition probability determine simulated burned fraction

**Fig. 6:**
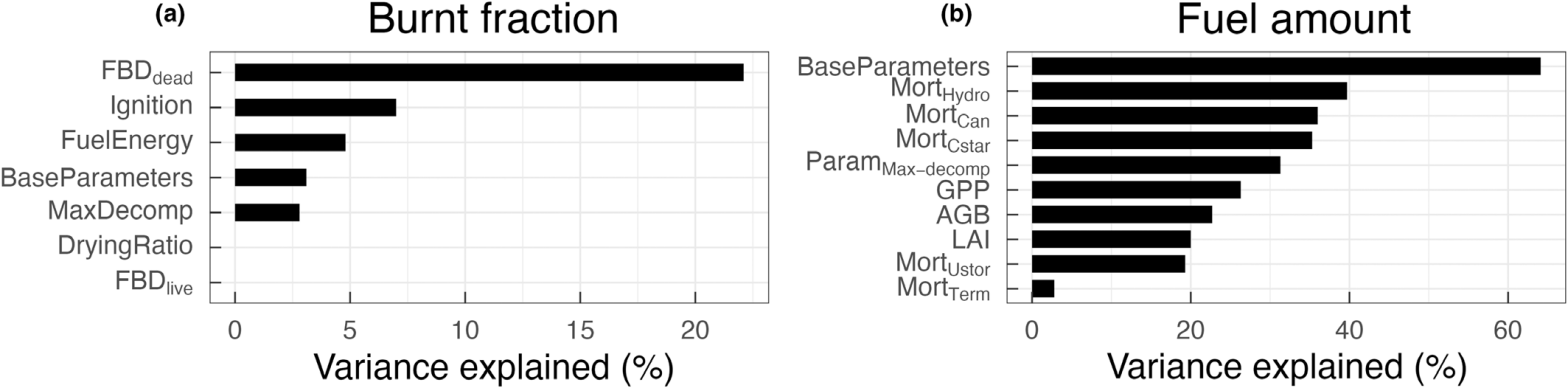
Top Community Land Model–Functionally Assembled Terrestrial Ecosystem Simulator model parameters and variables influencing simulated burned fraction (a) and fuel amount (b). FBD_dead_, fuel bulk density for dead leaf fuel; MaxDecomp, maximum decomposition rate; FBD_live_, fuel bulk density for live grass fuel; Base parameters refer to the eight base parameter sets selected from the fire‐off simulations; Mort_Hydro_, Mort_Cstar_, and Mort_Term_ refer to mortality caused by hydraulic failure, carbon starvation, and termination respectively; Mort_Can_ and Mort_Ustor_ refer to canopy‐ and understory‐layer mortality.

Simulated burned fraction (0.0003 ± 0.0007 yr^−1^ across all simulations) at the site was mainly determined by bulk density of dead leaf fuels with a negative correlation between the two in the model (Figs 6a, S4). Ignition density and fuel energy were among the top three model parameters determining grass fire burn fraction in CLM–FATES, with both positively influencing fire area. In the model formulation, fuel abundance is a major driver of burned fraction; therefore, we further examined the effects of both model parameters and variables on simulated fuel amount. The most influential factor was the selected PFT parameter set that varies in 20 biophysical traits, which explained > 60% of the total variance in simulated fuel amount (Fig. 6b). As expected, maximum litter fragmentation rate was also an important model parameter determining fuel availability at the time of fire. Model variables that had direct effects on litter influx, namely all of the mortality‐ and productivity‐relevant variables such as GPP, LAI, and AGB, were also among the top model variables to determine simulated fuel amount.

More than 80% of 500 ensemble members were able to reconstruct observed GPP, LAI, AGB, and energy fluxes at the site after fire was enabled in our simulations (Figs S5–S9). Six ensemble members had > 50% of years during 2000–2020 fall within 15–85% quantiles of observed mean annual burned fraction (Fig. S10). We chose three high‐performing ensemble members from the GENL group to further assess model performance at the regional scale. These three ensemble members were selected because: while fuel loads were overestimated across all ensemble members, the simulated mean fuel load of the three (0.54 ± 0.21 kg C m^−2^) was closer to observed fuel load (0.43 ± 0.17 kg C m^−2^) in tallgrass prairies (Kidnie & Wotton, 2015), but still higher than that in central coastal grasslands in California (0.004–0.35 kg C m^−2^, Ratcliff *et al*., 2022); and they represented a low, medium, and high annual burned fraction that best captured the range of observed annual burned fraction.

### The spatial correlation between productivity and burned area in California annual grasslands

**Fig. 7:**
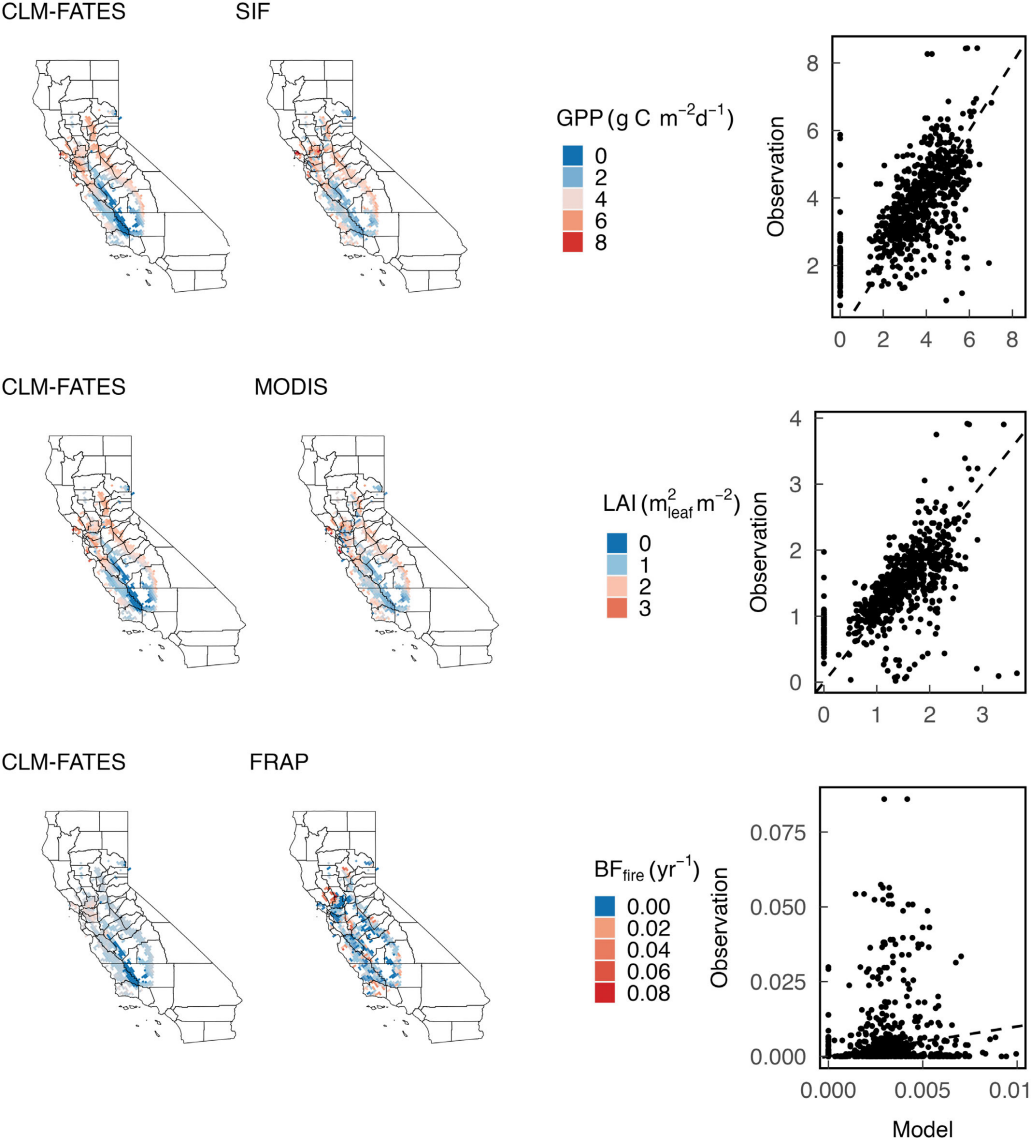
Model performance in capturing the spatial pattern in grassland productivity, structure, and fire dynamics. Community Land Model–Functionally Assembled Terrestrial Ecosystem Simulator simulated gross primary productivity, leaf area index (LAI) and burned fraction (*A*_Burnt_) in comparison with MODIS GPP and LAI and FRAP burned fraction across the annual grassland domain in California. Dashed lines are 1 : 1 lines.

**Fig. 8:**
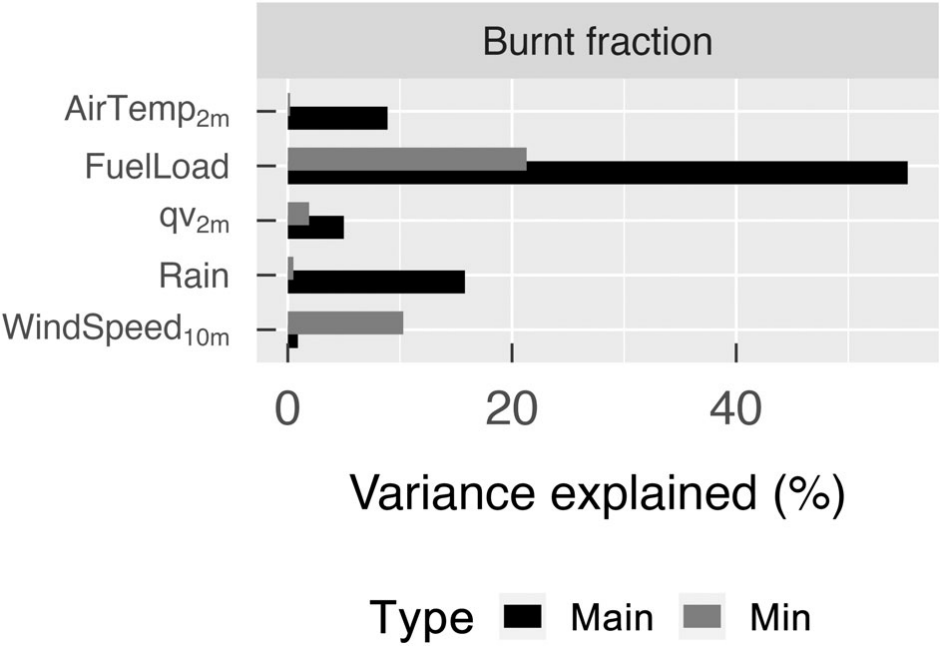
Correlated (main) and uncorrelated (min) effects of each meteorological driver and fuel load on simulated burned fraction (b). The correlated effect is calculated by including only the corresponding driver as single independent variable in a linear regression model and calculating the % variance explained by the driver; the uncorrelated effect is calculated by first accounting for effects of all of the other meteorological drivers and then building a linear regression model using the target meteorological driver as an independent variable and model residuals as dependent variable and calculating % variance explained. Notice that wind speed becomes the most important meteorological driver influencing burned fraction after accounting for the fuel load effect, while the effect of precipitation (Rain) decreases. AirTemp_2m_, 2 m air temperature; qv_2m_, 2 m specific humidity.

CLM–FATES, using the site‐level filtered parameter sets, was able to capture the spatial heterogeneity in peak growing season (March–May) GPP (RMSE: 1.27 g C m^−2^ d^−1^) and LAI (RMSE: 0.52 m^2^ m^−2^) across California annual grasslands, but tended to either overestimate or underestimate burned fraction (RMSE: 0.012 yr^−1^) in most regions. Both model and remote sensing estimates showed higher LAI and GPP in northern California and in the foothills of the Sierra Nevada Mountains relative to elsewhere in the state (Fig. 7). Toward the south, as mean annual precipitation decreases, both the simulated and remotely sensed estimates of LAI and GPP decreased. However, the mean burned fraction from the three regional simulations failed to fully capture the spatial pattern observed from remote sensing products: The ensemble member with a high annual burned fraction at the site level overestimated fire area in most grassland regions while the other two ensemble members underestimated burned fraction (Figs 7, S11). While the spatial pattern of simulated burned fraction coincided with the spatial heterogeneity in GPP, thus suggesting a fuel load effect (Fig. 8), observed mean burned fractions were positively skewed, with most grid cells having zero or low annual burned fraction and few grid cells having extremely high annual burned fraction. Sensitivity analysis also demonstrated that wind speed is the most influential factor for simulated burned areas after accounting for the fuel load effect (Fig. 8).

## Discussion

We examined the effects of alternate allometry assumptions on matter and energy exchange in a C_3_ annual grassland in California using model simulations. While simulated GPP, LAI, AGB, and sensible heat flux varied among the three allometry groups (Fig. 2), the effects of canopy architecture and carbon partitioning strategy differ from our expectations. *A. barbata* has the highest leaf‐to‐stem biomass ratio and was expected to result in a more productive community as suggested by previous work (Lane *et al*., 2000). However, the larger canopy area of *A. barbata*, combined with the higher leaf biomass per plant, can also result in stronger competition for light and space, and higher water demand due to greater canopy evapotranspiration at the individual plant level (Pretzsch *et al*., 2012). Given that water is one of the limiting factors in semiarid grasslands, it is likely that this large, leafy canopy of *A. barbata* led to reduced plant density and biomass per unit land area due to higher mortality. The effect of canopy architecture dominated over the effect of carbon partitioning strategies in the model to result in a lower mean ecosystem level LAI, GPP, and AGB for the AVBA ensemble compared with the other two ensembles. Related, lower biomass per unit crown area could result in greater sensible heat flux due to lower LAI and transpiration at the site scale and therefore lower latent heat flux. The AVBA ensemble also has a mean canopy cover of 0.477 m^2^ m^−2^, which is slightly higher than the BRDI (0.448 m^2^ m^−2^) and the GENL (0.446 m^2^ m^−2^) ensembles. This difference in canopy cover might also contribute to the variation in simulated sensible heat flux through albedo effects. It is worth noting that FATES is designed for closed‐canopy ecosystems so that canopy architecture‐mediated light and space competition might be overly emphasized in the model, with a strong influence on ecosystem structure. Competition for limiting soil resources (e.g. nutrients and water) can be relatively more important determinants of ecosystem structure and function in open ecosystems such as semiarid grasslands. However, in the current implementation of CLM–FATES, greater root biomass does not directly result in greater water or nutrient uptake. If allocating greater carbon to roots to actively compete for more water would actually be more beneficial for promoting ecosystem productivity than withstanding low water availability via a narrower crown in semiarid grasslands, allometry effects may differ from what we observed here. Empirical work is needed to help better understand how morphological variation among grassland species affects ecosystem structure and plant sensitivity to changing environments.

The behavior of the different allometries overlapped substantially at the site level, such that all three allometry groups had ensemble members well representing the observed site characteristics (Fig. 5). However, the model best captured the spatial heterogeneity in grassland LAI when using the generalized grass allometry instead of the *B. distachyon* allometry (Fig. S12). Thus, while either species‐specific or generalized C_3_ annual grass allometry can reconstruct community structure and ecosystem function at the site level, the latter is preferred at a larger spatial scale to represent the average growth curves of the C_3_ annual grass functional type. The differences in simulated regional LAI between the two allometries might result from the more varied species composition at the regional scale (Keeler‐Wolf *et al*., 2007) such that a general ecosystem‐scale pattern of growth and development can emerge.

As expected, leaf traits and SWC_Phen_ that influence phenology determine the simulated carbon, water, and energy cycling and their seasonal variations at the site; and selected best‐performing parameterizations use a combination of plausible trait values, varying within the range of 0.032–0.059 m^2^ g^−1^ C, 56.15–83.85 μmol CO_2_ m^−2^ s^−1^, and 0.124–0.169 m^3^ m^−3^ for SLA, $V_{c_{\max}}$, and SWC_Phen_, respectively. The importance of SLA and $V_{c_{\max}}$ in influencing LAI and GPP has been well‐documented in both land models and empirical studies (Wolf *et al*., 2006; Castanho *et al*., 2013; Ricciuto *et al*., 2018; Koven *et al*., 2020). Consistent with empirical studies at the site (Baldocchi *et al*., 2004; Xu & Baldocchi, 2004), seasonal dynamics in matter and energy exchange are influenced by change in soil volumetric water content (Figs 3, 4). The observed seasonal pattern in carbon flux is also a result of the seasonal variations in leaf traits (Ma *et al*., 2011). Given that our simulations do not incorporate a daylength effect on $V_{c_{\max}}$ and so are not resolving temporal or spatial variations in grass biophysical traits, the mismatch in GPP between model and data, especially at the regional scale, could be partially due to the lack of trait acclimation or adaptation to changing environments (Verheijen *et al*., 2015; Sterck *et al*., 2016). While leaf traits and SWC_Phen_ alternately influence simulated GPP and LAI as the grass phenological phase changes, the latter has a constant strong effect on simulated latent heat flux (Fig. 4). This could be due to the fact that SWC_Phen_ determines the length of growing season, thus the population size and structure that directly affect evapotranspiration.

The simulated AGB showed little sensitivity to the examined model parameters and a larger deviation from site observations for the selected parameter sets (Fig. 5). Aboveground biomass is a result of the interaction between factors including growth, mortality, and regrowth following disturbance, which thus may respond nonlinearly to change in individual model parameters. The large model‐data discrepancy in AGB is likely because site observations include both dead and live AGB given that observed AGB peaks in June while live AGB in California annual grassland usually peaks in April or May before onset of the dry season. This hypothesis is supported by a smaller model‐data discrepancy when comparing model simulated mean annual AGB to observations from another C_3_ annual grassland located in the Sierra Nevada Mountain foothills (Fig. S13, Seabloom *et al*., 2021).

Simulated fuel load and burned fraction varied significantly and outside the range of observations: Both AVBA ensemble members and two of the three BRDI ensemble members had much higher fuel load (fuel load > 1.0 kg C m^−2^) and burned fraction than the other four ensemble members (Fig. S14). The PFT parameter bases, which differ across 20 model parameters that are relevant to key plant life events, have the most influence on simulated fuel amount (Fig. 6b). Plant traits including $V_{c_{\max}}$ and SLA and key ecological processes such as litter decomposition rate, plant sensitivity to change in soil water content (e.g. SWC_Phen_), and drought mortality play important roles in affecting fuel load given their strong effects on ecosystem productivity and litter accumulation (Figs 3, 6).

The addition of further processes might also be necessary for accurately representing fuel load in annual grasslands. An annual life history is a drought adaptation strategy in regions with pronounced dry seasons (Volis *et al*., 2002; Kooyers, 2015; Monroe *et al*., 2019). The coupling of cooler temperature and ample precipitation in California's wet season favors the rapid growth and seed production in cool season, C_3_ annual grasses, which then become completely senescent by early‐ and mid‐summer; the whole population dies shortly after soil water content drops below a threshold (Holmes & Rice, 1996). We were able to capture these phenological changes by configuring annual grasses with a drought‐deciduous phenology and increasing drought‐induced mortality, which emphasizes the importance of both leaf‐ and organism‐level plant traits in governing the seasonal cycles of ecosystem fluxes in these annual grasslands. However, the current phenology model considers soil water content as the only control for plant green‐up and senescence, which might be oversimplified. Recent work suggests that photoperiod, in addition to soil water potential, can be another factor influencing annual grass phenology (Bart *et al*., 2017), indicating the potential need for a more mechanistic and integrated annual plant phenology model. In our CLM–FATES simulations, recurrent recruitment, including new recruits from seed following plant mortality and regrowth after leaf shedding before the seed carbon pool and plant carbon storage are depleted can result in excess carbon transfer to the litter pool in the model, contributing to an unreasonably high fuel load for grass‐dominant ecosystems. Thus, addition of environmental constraints on seed germination and regrowth may help reduce recruitment and allow a dormant period until new plants emerge (Hanbury‐Brown *et al*., 2022).

Simulated burned fraction decreased with higher fuel bulk density, and increased with greater fuel load. Empirical work shows that the negative effect of fuel bulk density on fire rate of spread – and thus burned area – in litter fuels is due to the slowed drying process and limited air flow when fuels are densely packed (de Magalhães & Schwilk, 2012). However, dead grass fuels often are standing canopy fuels with relatively low compactness (Brown, 1981; Hoffmann *et al*., 2012). Previous work argues that under a certain threshold, an increase in canopy fuel bulk density should increase spread rate due to increased fuel continuity when oxygen flow is not yet limited; and the correlation becomes negative once above that threshold (Kunst *et al*., 2012; Schwilk, 2015; Brou & Adou, 2022). These processes are not yet represented in the SPITFIRE‐derived CLM–FATES fire model. While the negative correlation between fuel bulk density and flame spread is generally observed (Catchpole *et al*., 1998), it is still surprising to see such a pattern in arid and semiarid grasslands, where fuels are relatively sparse. There are several potential explanations. First, our simulated fuel load is higher than observed grassland fuel loads, which might change simulated fire behavior. Second, we applied a wide range of fuel bulk densities for dead leaf fuels varying between 4 and 22 kg m^−3^, which is within the observed range for litter fuels but higher than that in standing grass fuelbeds (Kendall, 1979; Hoffmann *et al*., 2012; Kunst *et al*., 2012). Lastly, fire behavior in the current version of CLM–FATES was designed for surface fire that consumes ground litter fuels typical of forest ecosystems. The lack of canopy fuel characteristics and the resulting canopy fire behavior can lead to unrealistic fuel–fire relationships in regions that are not influenced by forest surface fires. To better simulate fire behavior, improvements in model structure are required to: separate dead grass fuels from litter fuels as they are different fuel types varying in fire behavior; incorporate canopy fuels, considering both standing grass fuels in grasslands and woody fuels in regions with shrub and tree cover; and capture canopy fire behavior.

In agreement with our hypothesis, simulated burned fraction follows the spatial pattern of grassland productivity at the regional scale, with higher mean burned fractions in the northern coastal regions and the Sierra foothills, and lower mean burned fractions in the southern regions. This regional pattern of burned fraction is thus largely determined by spatial heterogeneity in model meteorological drivers, such as precipitation and temperature, that directly affect fuel load (Fig. 8, the maximum effect and Fig. S15). However, after accounting for fuel load effects, wind speed becomes the most important meteorological variable influencing simulated fire area (Fig. 8, the minimum effect), supported by empirical work (Keeley & Syphard, 2019). In contrast to the strong spatial pattern in simulated burned fraction, observed fire area shows no clear relation to either vegetation productivity or climatic condition. Instead, observations generally indicate little or zero burned fraction in most of the grassland grid cells. The FRAP fire perimeter database only includes grass fires that are > 300 acres; this observation bias can contribute to overprediction of fire area by the model when compared to observations. However, the model‐observation discrepancy is still present when we compare simulated burned area to the Spatial Wildfire Occurrence data (Short, 2022), in which small grass fires are also included. In addition to potential observation bias, this large model‐observation discrepancy in burned area is also likely due to land management processes that are currently missing in the model: fire suppression, especially on managed grasslands; and grazing that removes significant amounts of fine fuels and creates a patchy fuel distribution that further suppresses fire spread across the landscape (Ratcliff *et al*., 2022, 2023). Moreover, we applied a constant ignition density across the model domain, which can cause over‐ and underprediction of fire depending on the actual ignition probability. A mechanistic ignition probability model, resolving the temporal and spatial variations in ignition density and accounting for anthropogenic causes will be useful for improving the model predicted fire regime (Syphard *et al*., 2017). Lastly, the high degree of stochasticity in observed fire also represents a challenge to improving model fidelity as compared to observed fire climatologies.

Our work contributes to limited prior progress on model development for grassy ecosystems. The adaptive dynamic global vegetation model (aDGVM), designed for semiarid tropical rangelands with an individual‐based framework, simulates grasses with dynamic carbon allocation (but only in proportional allocation to different carbon pools) and phenology schemes (Scheiter & Higgins, 2009). A further refinement of grass PFTs in aDGVM was made to distinguish between annual and perennial grasses by varying their carbon allocation and leaf traits, and applying different mortality mechanisms for the two (Pfeiffer *et al*., 2019). While encouraging, most models still simulate annual grasses as perennials despite the differences in their phenology, nutrient and water use efficiency, and carbon investment strategy that can influence ecosystem matter and energy exchange and fire regime (Taylor *et al*., 2010; Liu *et al*., 2019; Wilcox *et al*., 2023; Gao *et al*., 2024). Our work advances the modeling of grassy ecosystems in ESMs by implementing a data‐informed C_3_ annual grass that was thoroughly calibrated to reconstruct the seasonal variations and regional patterns in annual grassland productivity and structure, as well as some aspects of fire behavior. To facilitate future model simulations in grassy ecosystems, we implemented the new grass allometry into the current version of FATES and updated relevant parameters. We also provided the calibrated nonallometry parameters as part of the data files that are associated with this work (see 1993, 2018 section). Moreover, the observed allometry effect on ecosystem structure and function provides insights into understanding community assembly via species variations in morphology, which requires further empirical evidence.

### Conclusion

Our study highlights the importance of using observed plant allometry and traits in model parameterization, rather than ‘default’ parameter values, to reconstruct site‐level carbon, water, and energy fluxes and spatial heterogeneity in grassland GPP and LAI. We also found that grass allometry determines ecosystem structure and functioning via canopy‐architecture‐mediated space and resource competition. The seasonal dynamics in matter and energy exchange in California annual grasslands is affected by leaf physiological traits, plant phenology, and mortality in the model, underscoring the close coupling between physiology, phenology, and mortality that is needed to capture the seasonal cycles of annual grasses. Future model developments to further improve seasonal dynamics of biomass and thus fuel dynamics include implementing tissue senescence that downregulates photosynthesis and new leaf production. The large deviation between model simulated and observed fire area underlines the need to incorporate the human dimension into future fire model development, including not only the effects of land management on fuel characteristics and wildfire dynamics but also how human activity will affect ignition probability and wildland firefighting. A standard protocol and collaborative platform for real‐time monitoring of fire behavior and fire effects at the global scale is also needed to facilitate model development and validation, so accurate predictions of global wildfire dynamics are possible.

## Competing interests

None declared.

## Author contributions

XG, CDK and LMK led the design of the research with inputs from ML, CX, PT and ZR; AH and SR provided high‐resolution climate data for accomplishing the regional simulations; SL helped with model infrastructure to enable the use of the high‐resolution climate drivers for regional simulations; XG led the performance of the modeling experiments, data acquisition and analysis, and interpretation with input and feedback from CDK, LMK, ML, CX, PT and ZR; XG wrote the first draft of the manuscript with CDK, LMK, ML and ZR contributing to edits and improvement of the manuscript.

## Disclaimer

The New Phytologist Foundation remains neutral with regard to jurisdictional claims in maps and in any institutional affiliations.

## Supporting information


**Fig. S1** Grass allometry applied in Community Land Model–Functionally Assembled Terrestrial Ecosystem Simulator.
**Fig. S2** Correlations between Community Land Model–Functionally Assembled Terrestrial Ecosystem Simulator variables and tuned model parameters.
**Fig. S3** Comparisons of model simulated monthly means of gross primary productivity using default vs best‐performing parameters to site observations.
**Fig. S4** Correlations between Community Land Model–Functionally Assembled Terrestrial Ecosystem Simulator variables and tuned model parameters shown for one fire‐on ensemble using the base parameter set selected from the generalized C_3_ annual grass allometry ensemble.
**Fig. S5** Seasonal variation of simulated gross primary productivity monthly mean for the eight fire‐on ensembles that use base parameters selected from the fire‐off perturbed parameter ensemble.
**Fig. S6** Seasonal variation of simulated leaf area index monthly mean for the eight fire‐on ensembles compared with site observations.
**Fig. S7** Seasonal variation of simulated aboveground biomass monthly mean for the eight ensembles compared with site observations.
**Fig. S8** Seasonal variation of simulated latent heat flux monthly mean for the eight ensembles compared with site observations.
**Fig. S9** Seasonal variation of simulated sensible heat flux monthly mean for all the eight ensembles compared with site observations.
**Fig. 10** Six fire‐on ensemble members that have > 50% of simulated annual mean burned fraction fall within 15–85% quantiles of observations for years 2000–2020.
**Fig. S11** Community Land Model–Functionally Assembled Terrestrial Ecosystem Simulator simulated gross primary productivity, leaf area index, and burned fraction using the 6_node_005‐task_008 base parameter set and the comparison to observations.
**Fig. S12** Model simulated leaf area index using base parameters from the *Brachypodium distachyon* allometry group (RMSE: ±0.55).
**Fig. S13** Community Land Model–Functionally Assembled Terrestrial Ecosystem Simulator simulated annual mean live aboveground biomass using the eight selected parameter sets in comparison with observed annual mean live aboveground biomass for a C_3_ annual grassland located in the lower foothills of Sierra Nevada mountains in California.
**Fig. S14** Model simulated fuel amount for the eight ensembles using base parameters selected from the fire‐off simulations.
**Fig. S15** Correlations between meteorological drivers, model variables, and burned fraction.
**Notes S1** Allometric relationships defined for annual grass plant functional types in Community Land Model–Functionally Assembled Terrestrial Ecosystem Simulator.
Please note: Wiley is not responsible for the content or functionality of any Supporting Information supplied by the authors. Any queries (other than missing material) should be directed to the *New Phytologist* Central Office.

## Data Availability

Model source code and parameter files for modeling experiments are openly available in Zenodo at doi: 10.5281/zenodo.10223552. Scripts for post‐processing model simulations are publicly available at: https://github.com/XiulinGao/FATES‐CA‐grassland‐exp.
