# Supplementary material for: California annual grass phenology and allometry influence ecosystem dynamics and fire regime in a vegetation demography model
Source: New Phytol. 2025 Jan 30;245(6):2480–95. doi: 10.1111/nph.20421 (PMC11840405; doi:10.1111/nph.20421)
Supplement: Supplementary file 1 — Fig. S1 Grass allometry applied in Community Land Model–Functionally Assembled Terrestrial Ecosystem Simulator. Fig. S2 Correlations between Community Land Model–Functionally Assembled Terrestrial Ecosystem Simulator variables and tuned model parameters. Fig. S3 Comparisons of model simulated monthly means of gross primary productivity using default vs best‐performing parameters to site observations. Fig. S4 Correlations between Community Land Model–Functionally Assembled Terrestrial Ecosystem Simulator variables and tuned model parameters shown for one fire‐on ensemble using the base parameter set selected from the generalized C3 annual grass allometry ensemble. Fig. S5 Seasonal variation of simulated gross primary productivity monthly mean for the eight fire‐on ensembles that use base parameters selected from the fire‐off perturbed parameter ensemble. Fig. S6 Seasonal variation of simulated leaf area index monthly mean for the eight fire‐on ensembles compared with site observations. Fig. S7 Seasonal variation of simulated aboveground biomass monthly mean for the eight ensembles compared with site observations. Fig. S8 Seasonal variation of simulated latent heat flux monthly mean for the eight ensembles compared with site observations. Fig. S9 Seasonal variation of simulated sensible heat flux monthly mean for all the eight ensembles compared with site observations. Fig. 10 Six fire‐on ensemble members that have > 50% of simulated annual mean burned fraction fall within 15–85% quantiles of observations for years 2000–2020. Fig. S11 Community Land Model–Functionally Assembled Terrestrial Ecosystem Simulator simulated gross primary productivity, leaf area index, and burned fraction using the 6_node_005‐task_008 base parameter set and the comparison to observations. Fig. S12 Model simulated leaf area index using base parameters from the Brachypodium distachyon allometry group (RMSE: ±0.55). Fig. S13 Community Land Model–Functionally Assembled Terrestrial Ecosystem Sim [file NPH-245-2480-s001.docx]

New Phytologist Supporting Information

Article title: California annual grass phenology and allometry influence ecosystem dynamics and fire regime in a vegetation demography model

Authors: Xiulin Gao, Charles D. Koven, Marcos Longo, Zachary Robbins, Polly Thornton, Alex Hall, Samuel Levis, Stefan Rahimi, Chonggang Xu, Lara M. Kueppers

Article acceptance date: 10 January 2025

**Notes S1. Allometric relationships defined for annual grass PFTs in CLM-FATES.** Details of grass allometry in CLM-FATES for aboveground biomass, leaf and fine root biomass, and plant height and crown area can be found below.

Aboveground stem biomass is a function of both plant height and diameter measured at the base of a grass, loosely based on Saldarriaga et al. (Saldarriaga et al. 1988), but ignoring the wood density effect. We also modified the allometry model so that there is no dead or live woody biomass for grass PFTs. The term “stem biomass” here only refers to aboveground non-leaf, non-woody structural biomass in grasses, which is classified as fine litter fuels after grass mortality. Leaf biomass is a function of basal diameter and is capped when the plant reaches its maximum height. Plant height is determined by basal diameter and capped at the observed maximum height for each of the three allometry groups (Fig. S1). Development of the grass crown area also responds to the change in basal diameter using a two-parameter power law function (Fig. S1). Lastly, fine-root biomass is proportional to leaf biomass with a scaling factor of one for all three allometry groups, supported by previous studies (Poorter et al. 2012, Gao et al. 2024).


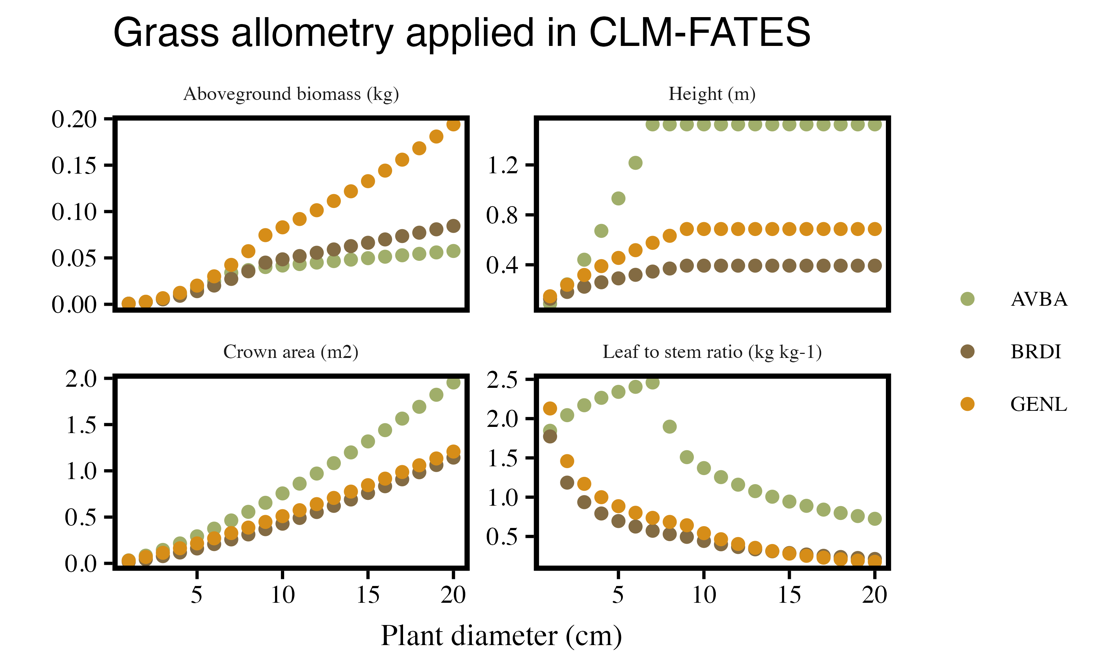


Figure S1. **Grass allometry applied in CLM-FATES.** Variations in plant canopy architecture (plant height and crown area), total aboveground biomass, and leaf to stem ratio for the allometry of *Avena barbata* (AVBA), *Brachypodium distachyon* (BRDI), and a generalized grass PFT in CLM-FATES. Note that AVBA is parameterized to be a tall grass with larger crown area while BRDI is parameterized to be a short grass with smaller crown area. The BRDI and GENL allometry are similar in canopy architecture and biomass partition between leaf and stem. Allometry parameterizations are based on empirical data by Gao et al. (2024).


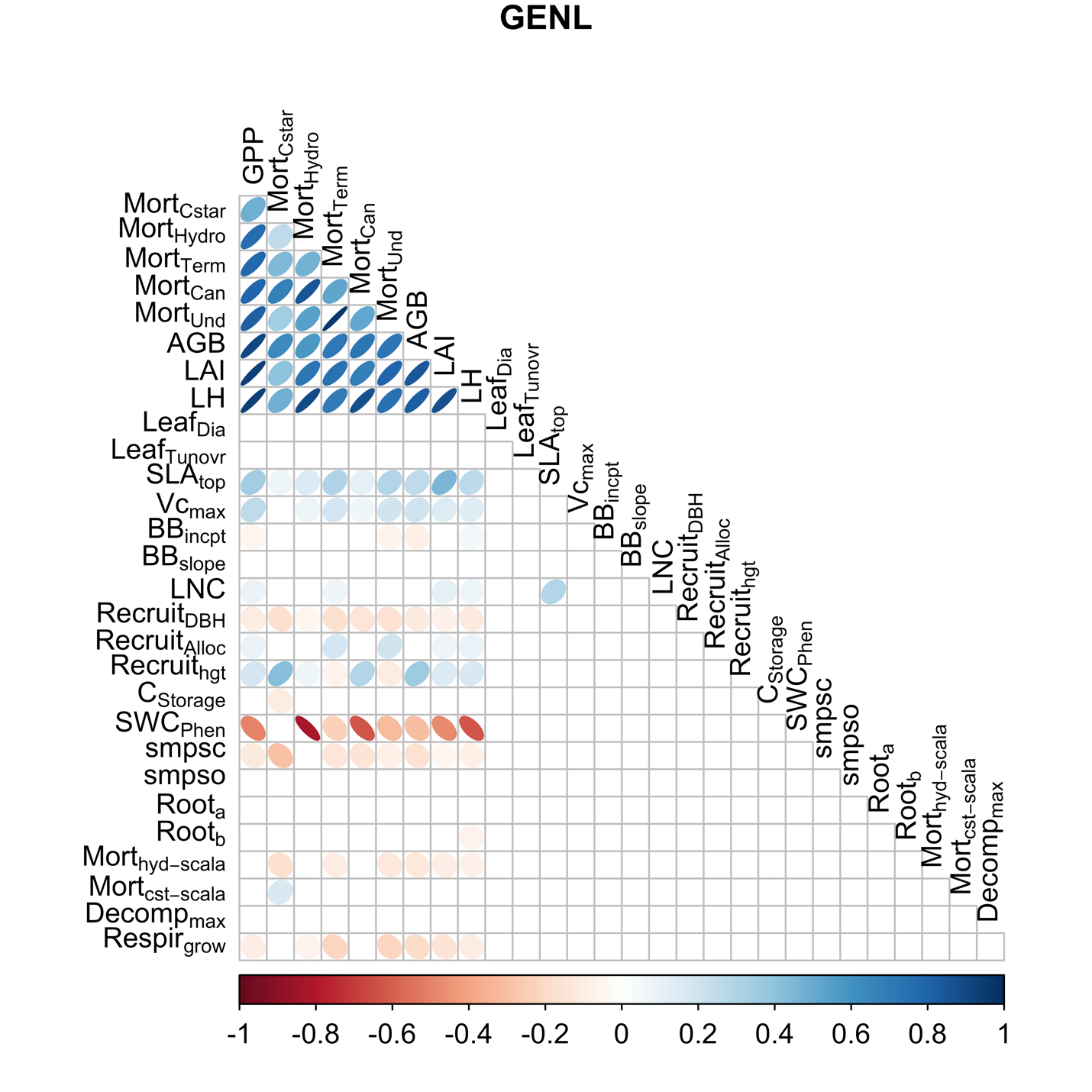


Figure S2. Correlations between CLM-FATES variables and tuned model parameters. Results are only shown for the generalized allometry ensemble, which are the same for the AVBA and BRDI allometry ensembles. Only significant (p < 0.05) correlations are shown in colored ovals. Mort_Cstar_ , Mort_Hydro_, Mort_Term_ : mortality due to carbon starvation, hydraulic failure, and termination respectively; Mort_Can_ and Mort_Und_: canopy-layer and understory-layer mortality respectively; AGB: aboveground biomass; LAI: leaf area index; LH: latent heat flux; Leaf_Dia_: leaf diameter; Leaf_Turnovr_: leaf turnover rate; SLA_top_: specific leaf area of canopy layer; Vc_max_: maximum leaf carboxylation rate at 25°C; BB_incpt_ and BB_slope_: Ball-Berry stomatal conductance intercept and slope respectively; LNC: leaf N:C ratio; Recruit_DBH_: threshold DBH size for reproduction; Recruit_Alloc_: carbon allocation to reproduction after reach the threshold DBH size; Recruit_hgt_: minimum height of new recruits; C_Storage_: proportion of carbon allocate to storage pool; SWC_Phen_: threshold soil water content triggering leaf-on and leaf-off; smpsc: threshold soil water matric potential triggering stomata closure; smpso: threshold soil water matric potential triggering stomata opening; Root_a_ and Root_b_: rooting profile parameter a and b; Mort_hyd-scala_ and Mort_cst-scala_: scalar factor for hydraulic failure and carbon starvation mortality respectively; Decom_max_: maximum decomposition rate; Respir_grow_: growth respiration.


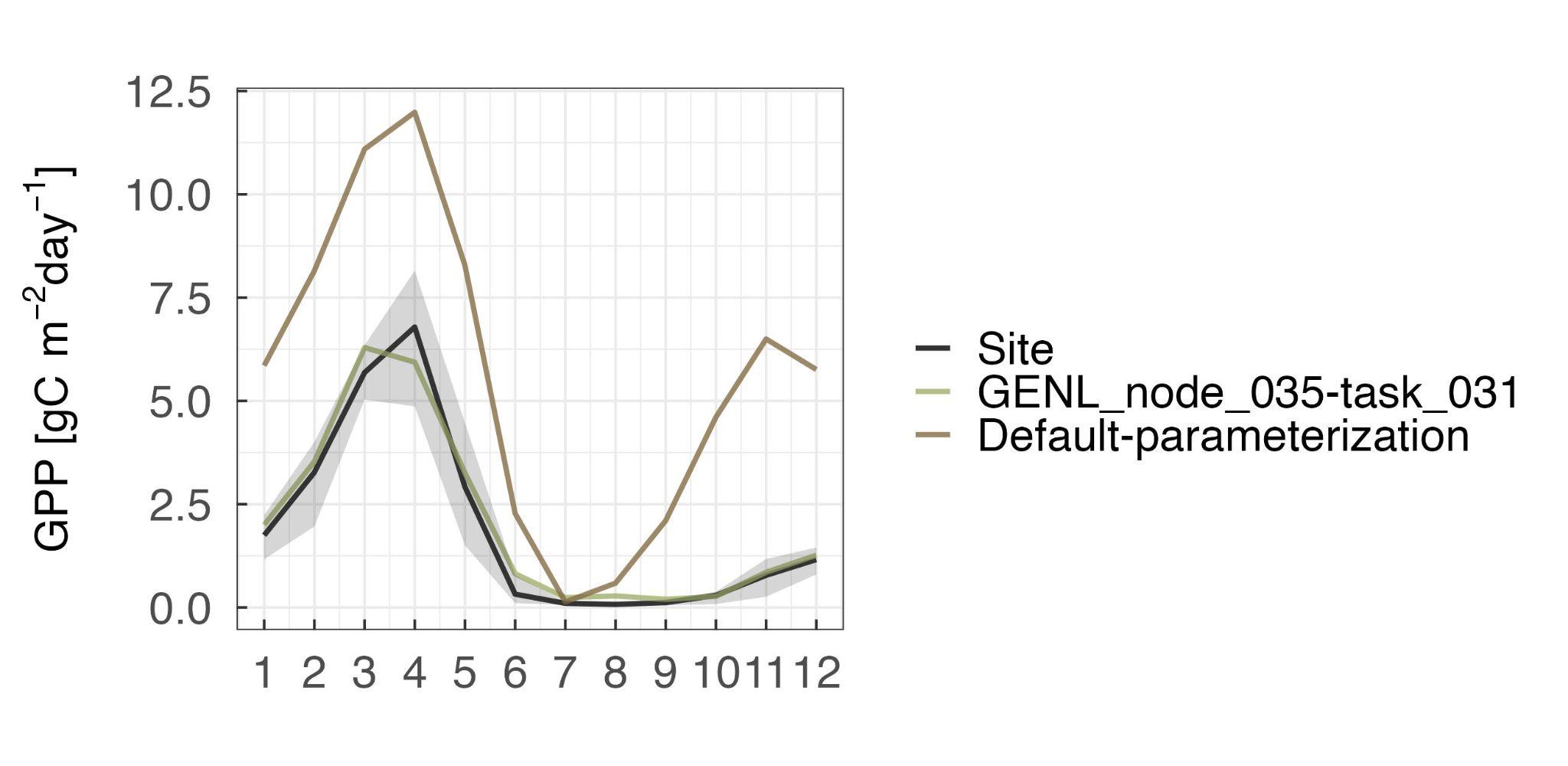


Figure S3. Comparisons of model simulated monthly means of GPP using default vs best-performing parameters to site observations. CLM-FATES simulated monthly means of GPP using default C_3_ grass parameterizations (Default-parameterization); and calibrated parameters from one of eight site-optimized parameterizations using the generalized grass allometry (GENL_node_035-task_031) in comparison to observations (Site) at Vaira Ranch. Fire is turned off in both simulations. Shaded area is the 15% - 85% quantiles of observations. Labels on the x-axis refer to the month of the year. Note the improvement in simulated seasonal GPP dynamics after we calibrated both allometry and non-allometry traits in the model. Also note the differences in GPP between observations and the default simulations during the senescent stage (June to October), which is due to the fact that default FATES does not have an annual-specific phenology or life history.


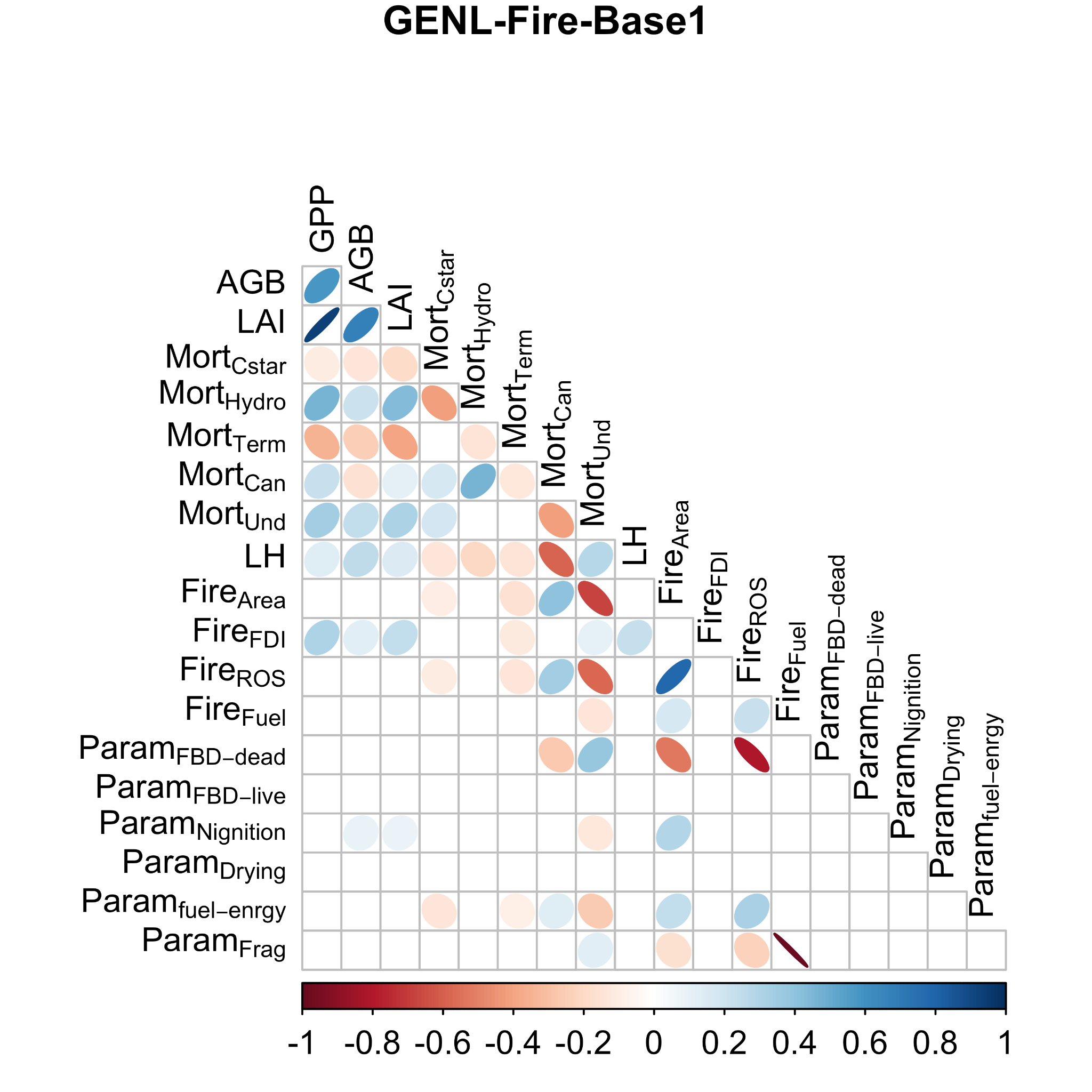


Figure S4: Correlations between CLM-FATES variables and tuned model parameters shown for one fire-on ensemble using the base parameter set selected from the generalized C_3_ annual grass allometry ensemble. Only significant correlations (p < 0.05) are shown. Fire_Area_: burned fraction; Fire_FDI_: fire danger index; Fire_ROS_: rate of spread; Fire_Fuel_: fuel load; Param_FBD-dead_: fuel bulk density of 1-hour litter fuel; Param_FBD-live_: fuel bulk density of live grass fuel; Param_Nignition_: lightning ignition density; Param_Drying_: parameter shapes the fuel drying curve; Param_fuel-energy_: fuel energy; Param_Frag_: maximum decomposition rate. Other abbreviations are the same as in figure S2.


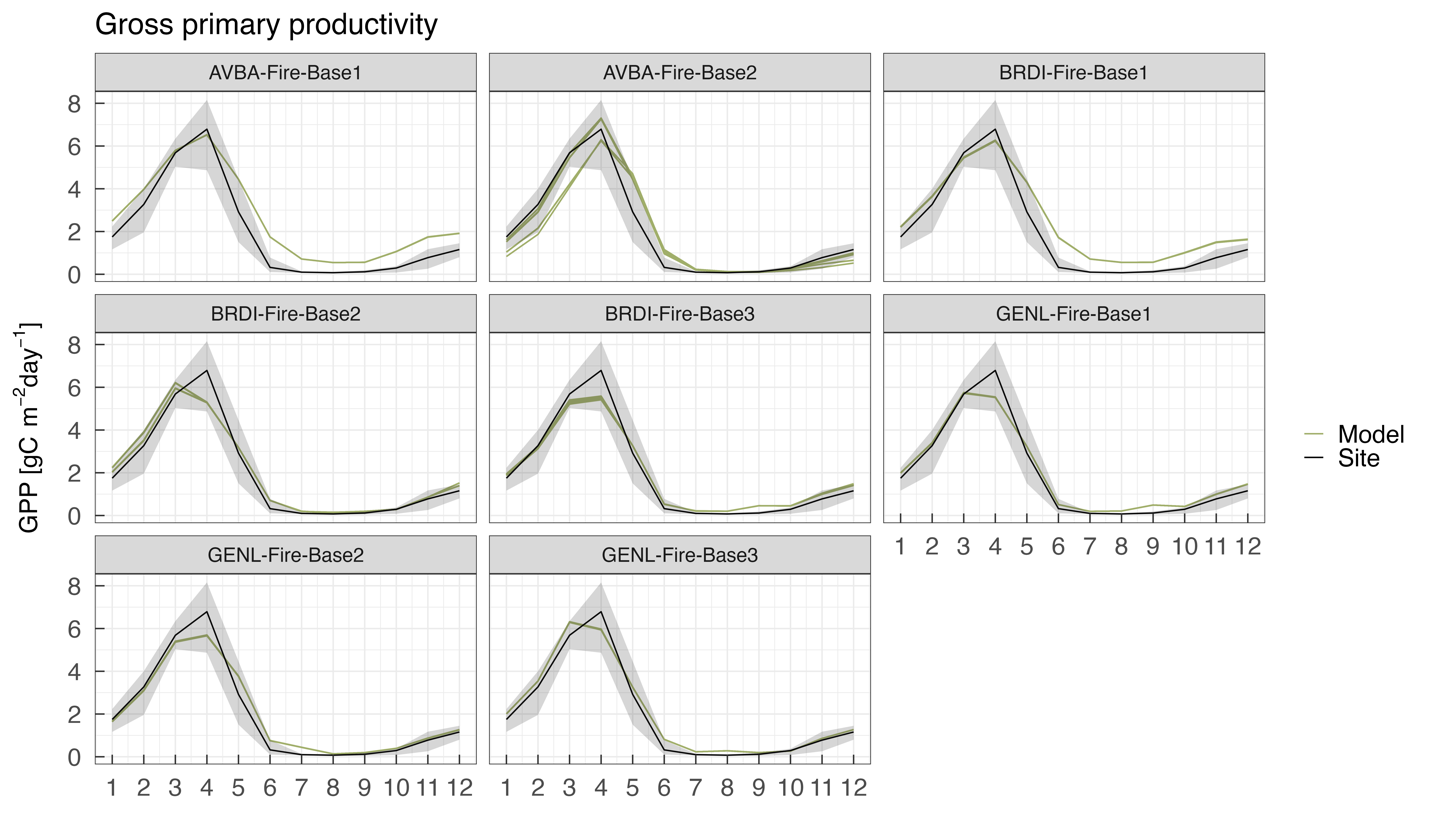


Figure S5. Seasonal variation of simulated gross primary productivity monthly mean for the eight fire-on ensembles that use base parameters selected from the fire-off perturbed parameter ensemble. Means of site observations are shown in black lines with the gray shaded area representing 15%-85% of observations. Model means are plotted for all 500 ensemble members and most of them overlap indicating little effect of fire parameters on GPP.


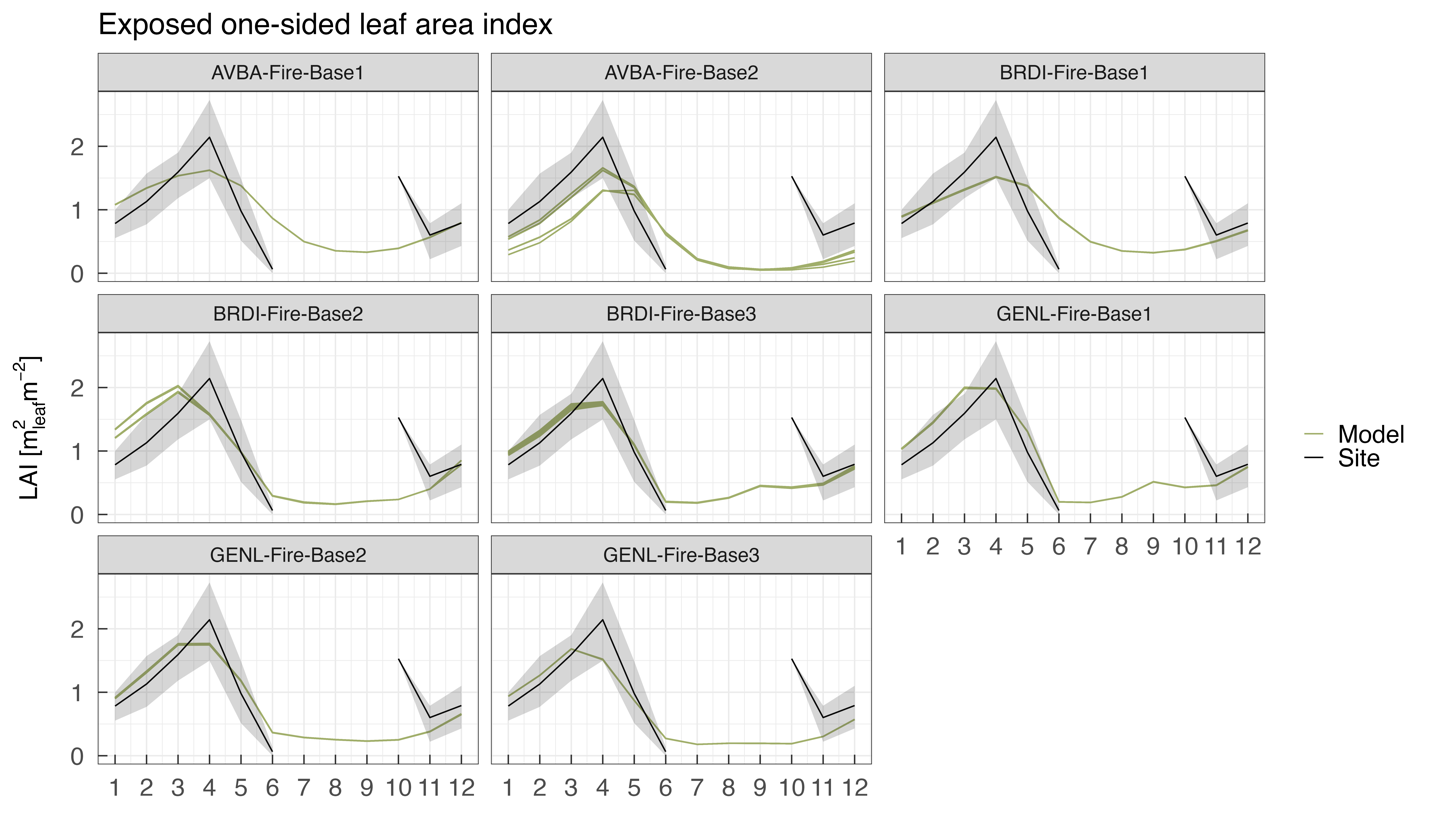


Figure S6. Seasonal variation of simulated leaf area index monthly mean for the eight fire-on ensembles compared to site observations. Same formatting as in Fig. S5.


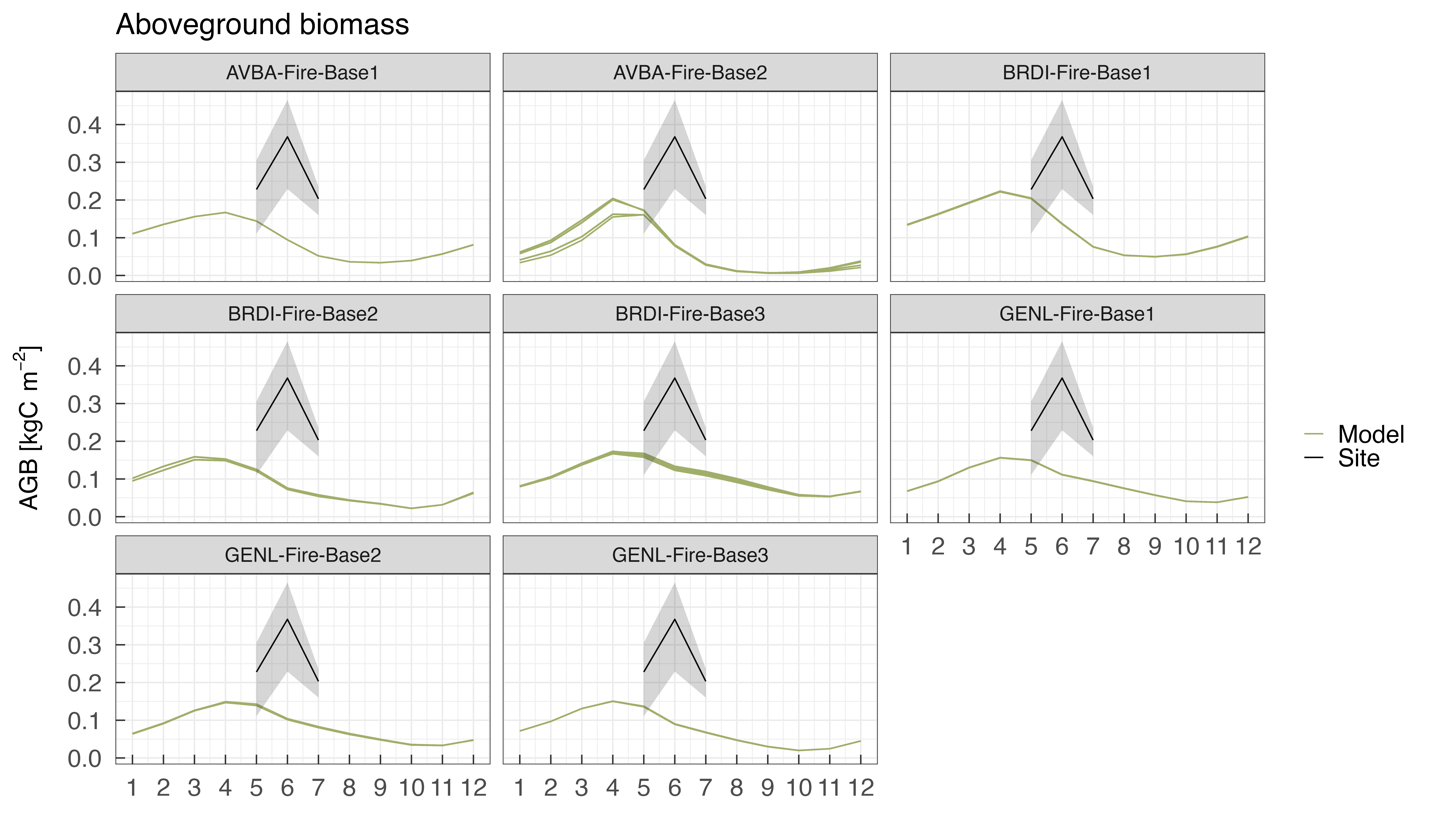


Figure S7. Seasonal variation of simulated aboveground biomass monthly mean for the eight ensembles compared to site observations. Same formatting as in Fig. S5.


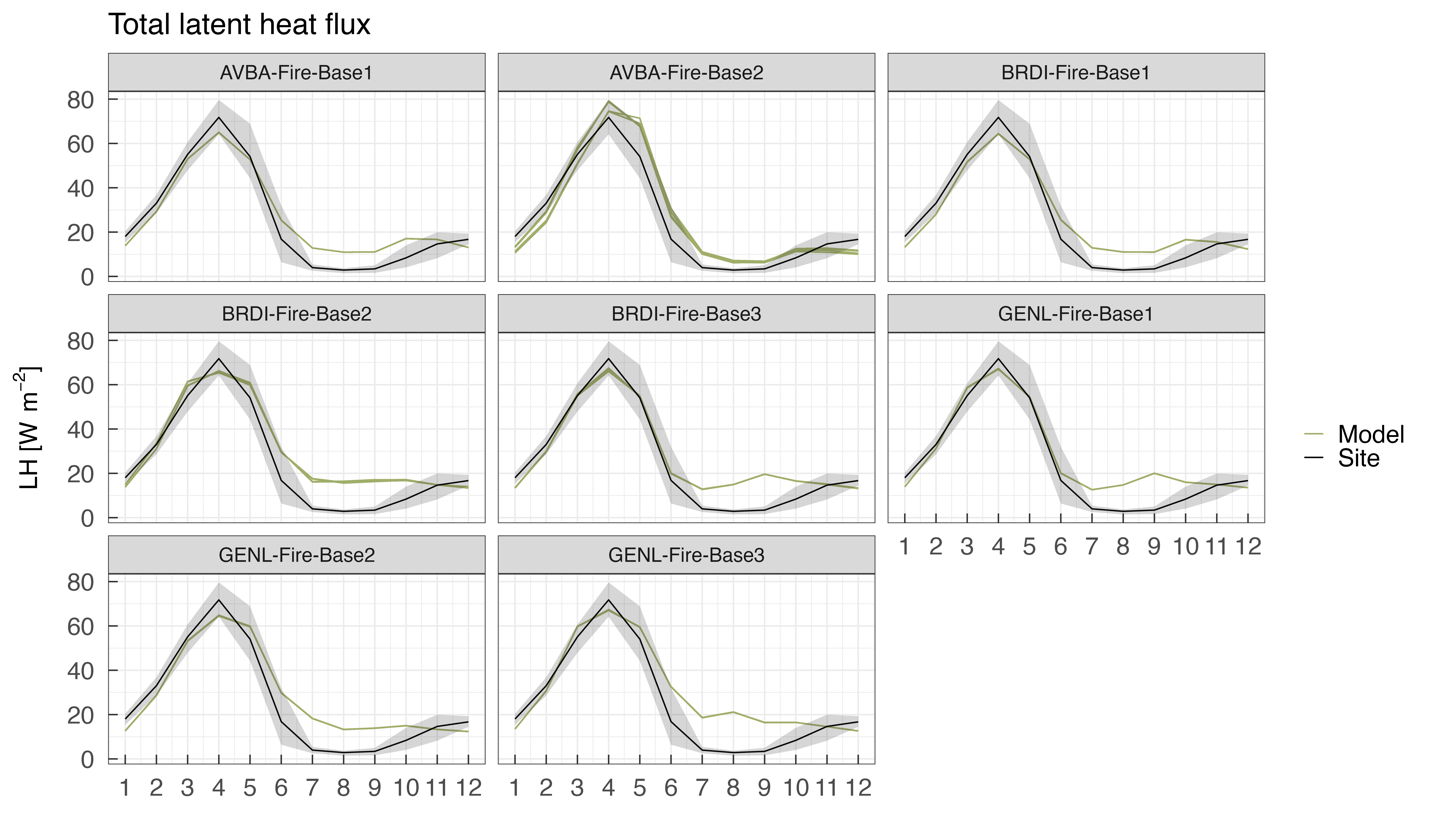


Figure S8. Seasonal variation of simulated latent heat flux monthly mean for the eight ensembles compared to site observations. Same formatting as in Fig S5.


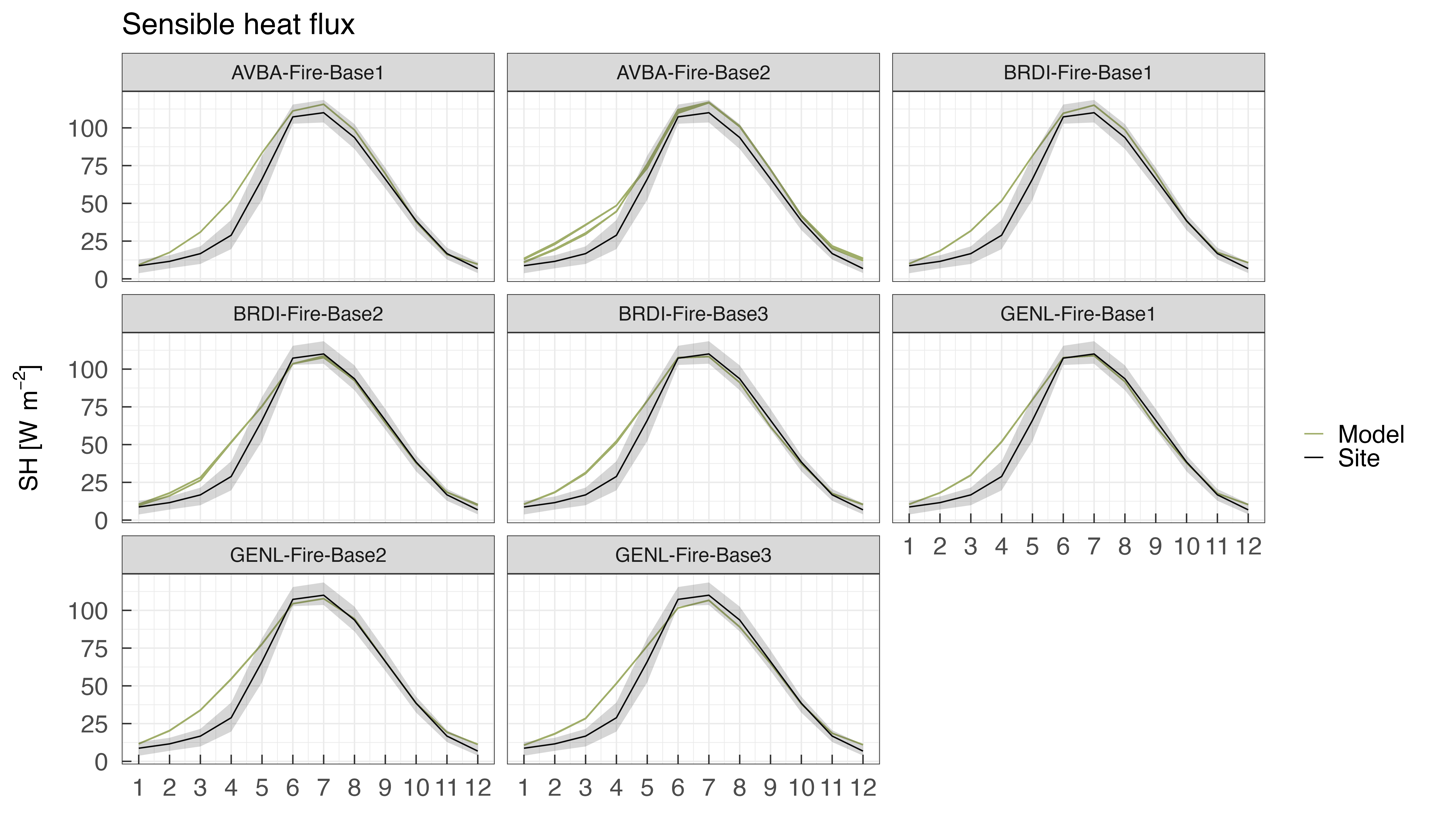


Figure S9. Seasonal variation of simulated sensible heat flux monthly mean for all the eight ensembles compared to site observations. Same formatting as in Fig. S5.


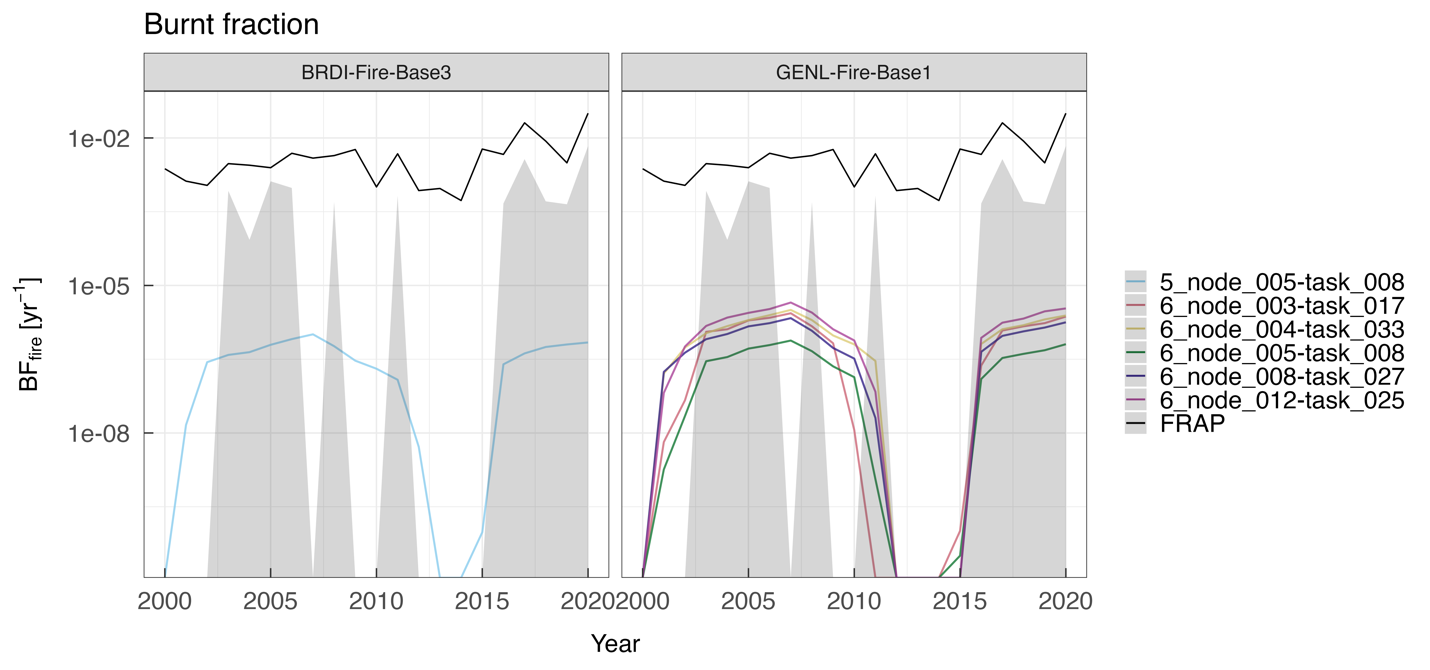


Figure S10. Six fire-on ensemble members that have more than 50% of simulated annual mean burned fraction fall within 15% - 85% quantiles of observations for years 2000-2020. Lines are ensemble member means, color-coded for ensemble member ID. Black lines are observed burned fraction averaged across California annual grassland region with shaded gray area representing 15% - 85% quantiles of observations. As FRAP data is positively skewed, the 15% - 85% quantiles of observation is much lower than the mean. We retained three ensemble members including 6_node_005-task_008, 6_node_008-task_027, and 6_node_012-task_025 for assessing model performance at the regional scale.


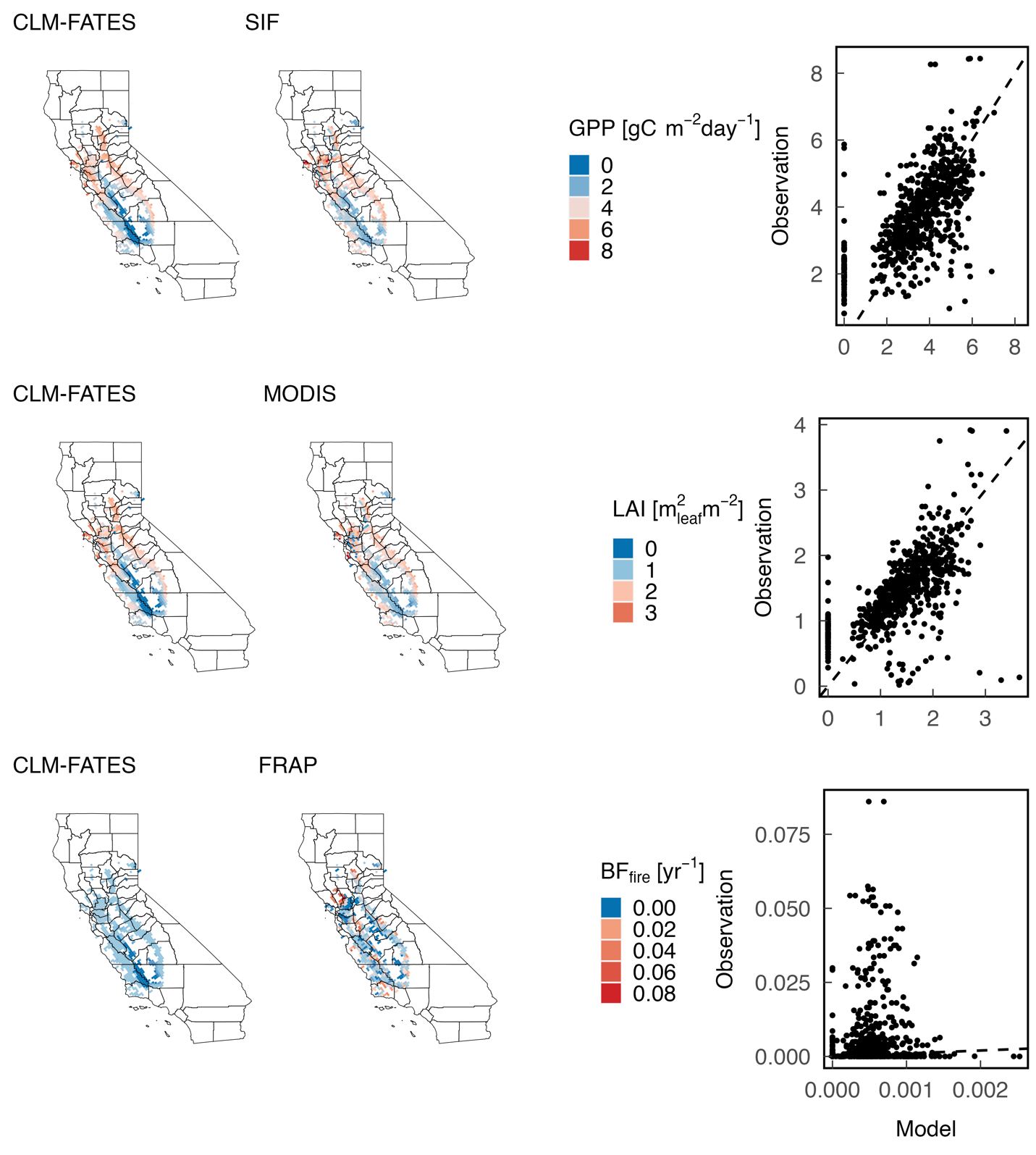


Figure S11. CLM-FATES simulated GPP, LAI, and burned fraction using the 6_node_005-task_008 base parameter set and the comparison to observations. Notice that when compared to Figure 7 (using the 6_node_012-task_025 base parameter set), there are no obvious differences between the two regional simulations in terms of GPP and LAI. CLM-FATES tended to underestimate burned fraction when using the 6_node_005-task_008 base parameter set, which also generated much lower burned fraction at the site level (see Figure S10).


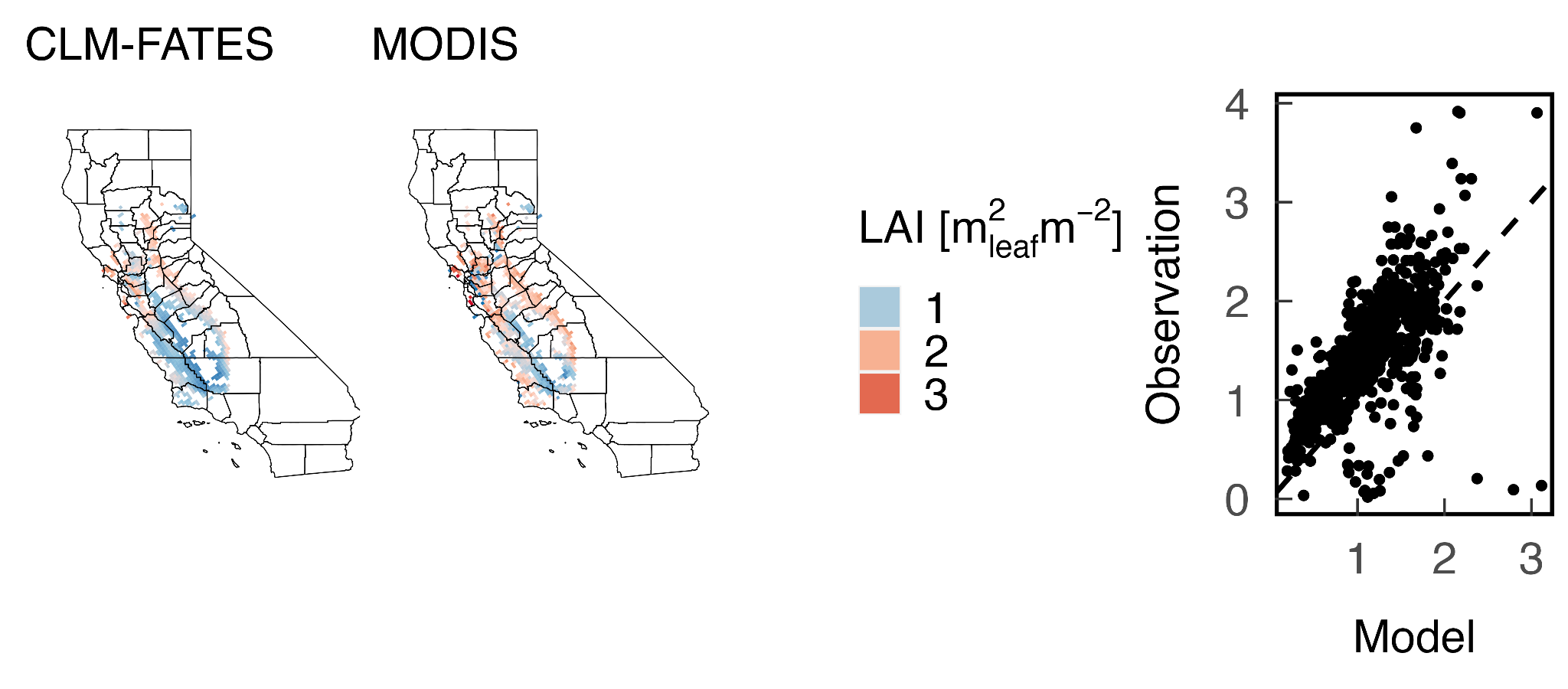


Figure S12. Model simulated leaf area index using base parameters from the BRDI allometry group (RMSE: ±0.55). Notice the underestimated LAI in the lower Sierras and the central coastal region, which is not observed for simulations using base parameters from the GENL allometry group (Fig. 7 middle panel).


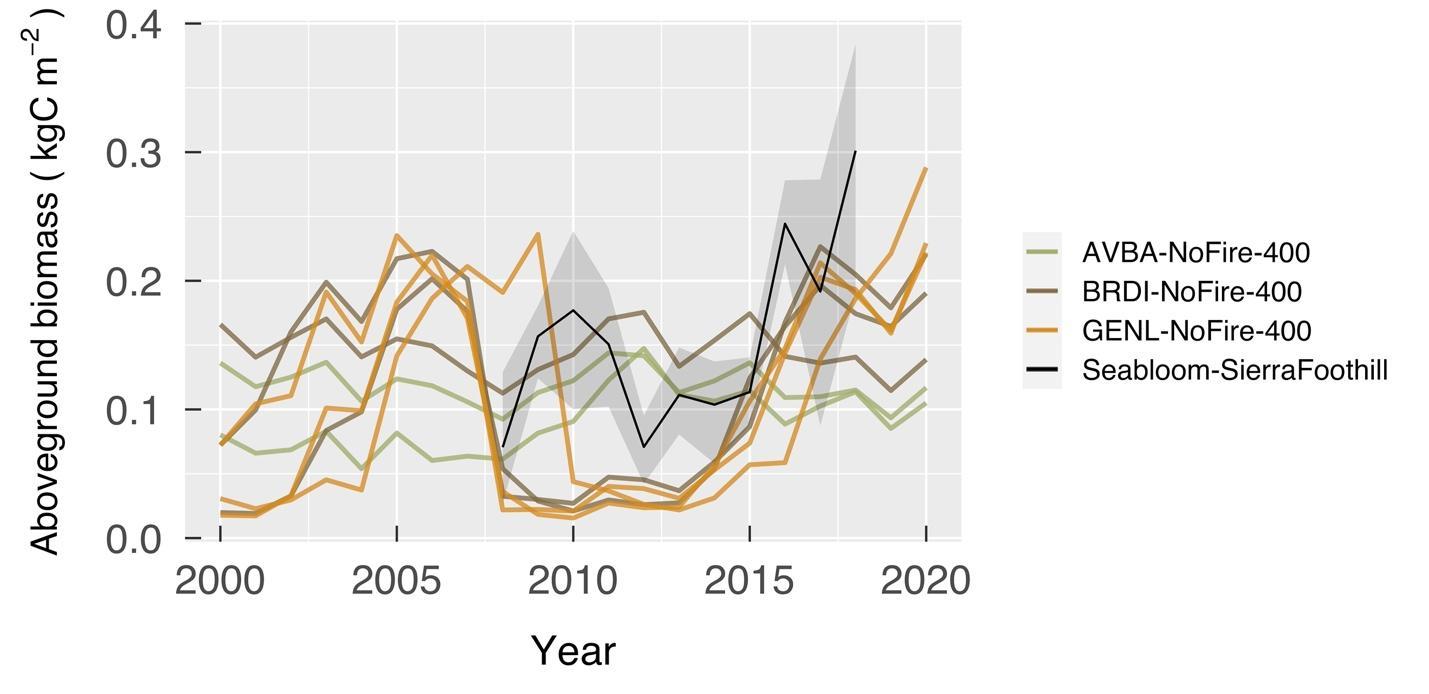


Figure S13. CLM-FATES simulated annual mean live aboveground biomass using the eight selected parameter sets in comparison to observed annual mean live aboveground biomass for a C_3_ annual grassland located in the lower foothills of Sierra Nevada mountains in California. Measurements are only available at annual scale so we cannot compare the seasonal variations of AGB. This comparison, however, indicates that CLM-FATES simulated live AGB is less likely to underestimate AGB for California annual grassland, which is in contrast to what is suggested by Fig.5c. Shaded area is the 15% - 85% quantiles of observations. Data is from the control plot measurements in Seabloom et al. (2021)


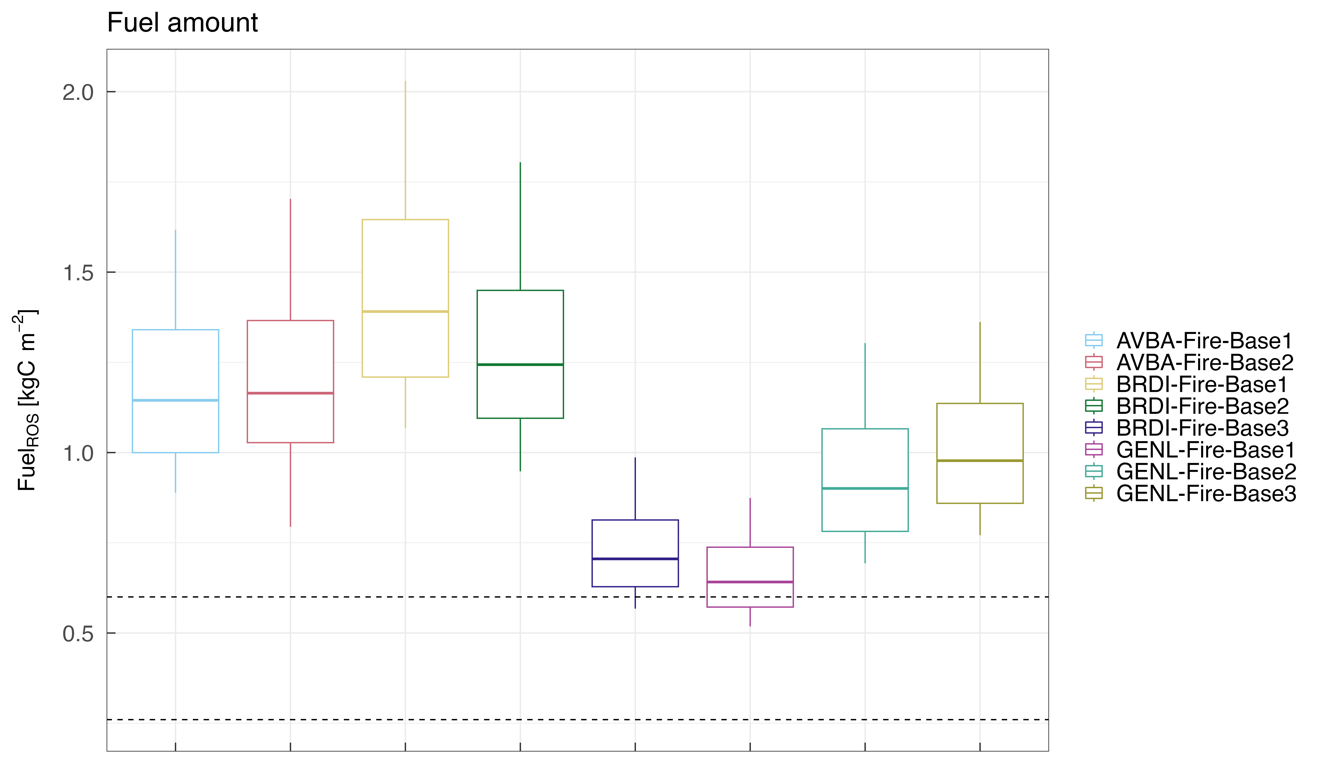


Figure S14. Model simulated fuel amount for the eight ensembles using base parameters selected from the fire-off simulations. The two dashed lines refer to the maximum (0.6 kg C m^-2^) and minimum (0.26 kg C m^-2^) fuel loads observed. To assess model performance at regional scale, we retained three ensemble members from the GENL-Fire-Base1 group given its relatively low fuel load that is closer to observed grassland fuel load.


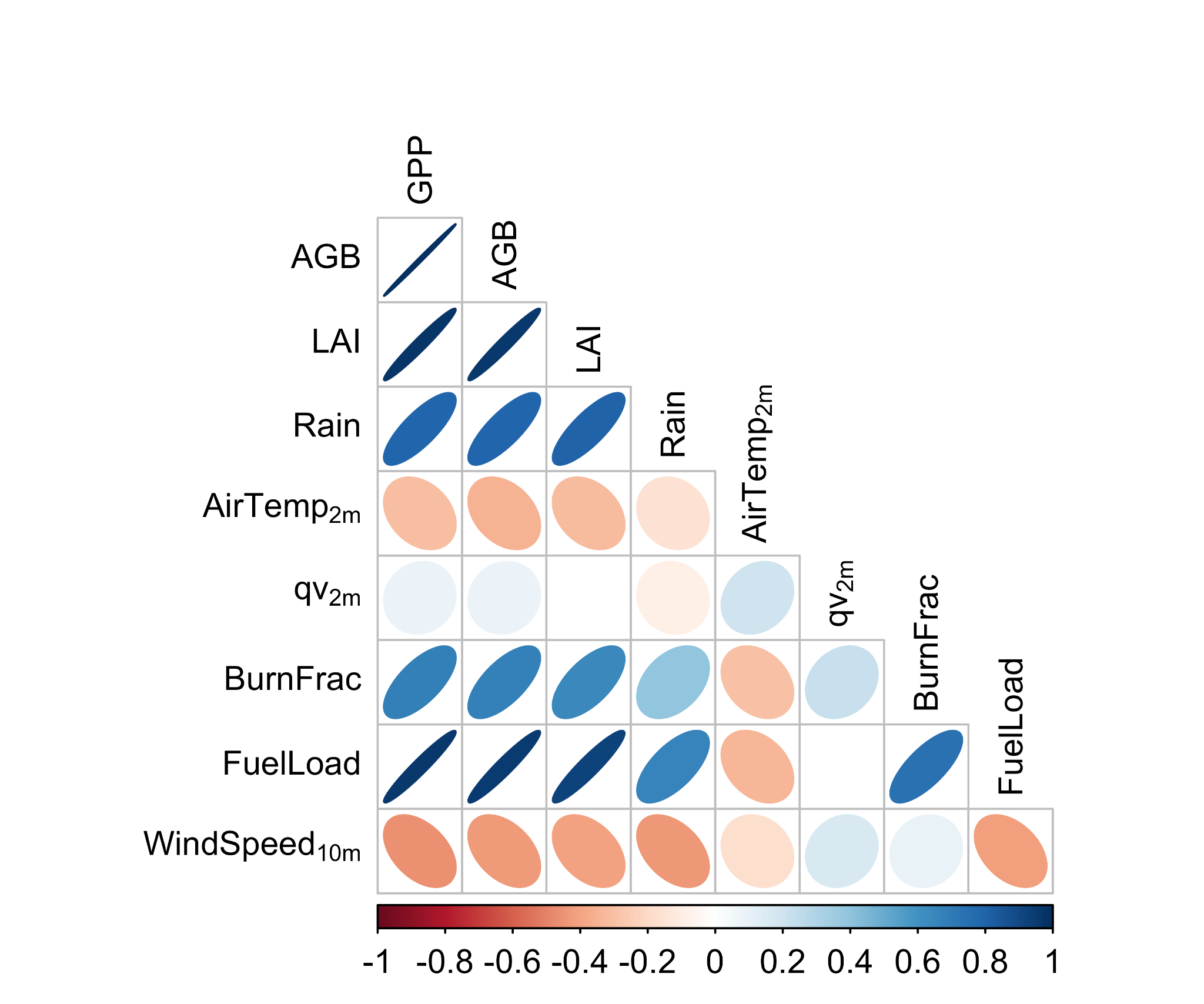


Figure S15. Correlations between meteorological drivers, model variables, and burned fraction. AirTemp_2m_: 2 m air temperature; qv_2m_: 2 m specific humidity.

References

Gao, X., C. D. Koven, and L. M. Kueppers. 2024. Allometric relationships and trade‐offs in 11 common Mediterranean‐climate grasses. Ecological Applications:e2976.

Poorter, H., K. J. Niklas, P. B. Reich, J. Oleksyn, P. Poot, and L. Mommer. 2012. Biomass allocation to leaves, stems and roots: Meta-analyses of interspecific variation and environmental control. New Phytologist 193:30–50.

Saldarriaga, J. G., D. C. West, M. L. Tharp, and C. Uhl. 1988. Long-Term Chronosequence of Forest Succession in the Upper Rio Negro of Colombia and Venezuela. Page Source: Journal of Ecology.

Seabloom, E. W., P. B. Adler, J. Alberti, L. Biederman, Y. M. Buckley, M. W. Cadotte, S. L. Collins, L. Dee, P. A. Fay, J. Firn, N. Hagenah, W. S. Harpole, Y. Hautier, A. Hector, S. E. Hobbie, F. Isbell, J. M. H. Knops, K. J. Komatsu, R. Laungani, A. MacDougall, R. L. McCulley, J. L. Moore, J. W. Morgan, T. Ohlert, S. M. Prober, A. C. Risch, M. Schuetz, C. J. Stevens, and E. T. Borer. 2021. Increasing effects of chronic nutrient enrichment on plant diversity loss and ecosystem productivity over time. Ecology 102.
